# Cell Cycle- and Cancer-Associated Gene Networks Activated by Dsg2: Evidence of Cystatin A Deregulation and a Potential Role in Cell-Cell Adhesion

**DOI:** 10.1371/journal.pone.0120091

**Published:** 2015-03-18

**Authors:** Abhilasha Gupta, Daniela Nitoiu, Donna Brennan-Crispi, Sankar Addya, Natalia A. Riobo, David P. Kelsell, Mỹ G. Mahoney

**Affiliations:** 1 Department of Dermatology and Cutaneous Biology, Thomas Jefferson University, Philadelphia, Pennsylvania, United States of America; 2 Center for Cutaneous Research, Blizard Institute, Barts and the London School or Medicine and Dentistry, Queen Mary University of London, London, United Kingdom; 3 Department of Biochemistry and Molecular Biology, Thomas Jefferson University, Philadelphia, Pennsylvania, United States of America; 4 Kimmel Cancer Center, Department of Cancer Biology, Thomas Jefferson University, Philadelphia, Pennsylvania, United States of America; University Hospital Hamburg-Eppendorf, GERMANY

## Abstract

Cell-cell adhesion is paramount in providing and maintaining multicellular structure and signal transmission between cells. In the skin, disruption to desmosomal regulated intercellular connectivity may lead to disorders of keratinization and hyperproliferative disease including cancer. Recently we showed transgenic mice overexpressing desmoglein 2 (Dsg2) in the epidermis develop hyperplasia. Following microarray and gene network analysis, we demonstrate that Dsg2 caused a profound change in the transcriptome of keratinocytes *in vivo* and altered a number of genes important in epithelial dysplasia including: calcium-binding proteins (S100A8 and S100A9), members of the cyclin protein family, and the cysteine protease inhibitor cystatin A (CSTA). CSTA is deregulated in several skin cancers, including squamous cell carcinomas (SCC) and loss of function mutations lead to recessive skin fragility disorders. The microarray results were confirmed by qPCR, immunoblotting, and immunohistochemistry. CSTA was detected at high level throughout the newborn mouse epidermis but dramatically decreased with development and was detected predominantly in the differentiated layers. In human keratinocytes, knockdown of Dsg2 by siRNA or shRNA reduced CSTA expression. Furthermore, siRNA knockdown of CSTA resulted in cytoplasmic localization of Dsg2, perturbed cytokeratin 14 staining and reduced levels of desmoplakin in response to mechanical stretching. Both knockdown of either Dsg2 or CSTA induced loss of cell adhesion in a dispase-based assay and the effect was synergistic. Our findings here offer a novel pathway of CSTA regulation involving Dsg2 and a potential crosstalk between Dsg2 and CSTA that modulates cell adhesion. These results further support the recent human genetic findings that loss of function mutations in the CSTA gene result in skin fragility due to impaired cell-cell adhesion: autosomal-recessive exfoliative ichthyosis or acral peeling skin syndrome.

## Introduction

Desmosomes are major adhesion structures localized to the cell-cell borders of epithelial cells where the cytoplasmic plaque components, including the plakin (desmoplakin) and keratin families, assemble with the armadillo (plakoglobin and plakophilins) and cadherin (desmogleins and desmocollins) protein families [1,2]. These adhesion structures are essential not only for the maintenance of cell structure and integrity, but also for tissue development and morphogenesis. Mutations within the desmosome are the underlying cause of many skin fragility disorders with or without heart abnormalities [3]. Additionally, desmosomes also serve as “signaling centers” playing an active role in modulating several important pathways, including the Wnt/β-catenin and the T-cell factor/lymphoid enhancer factor [4]. Mounting evidence supports their participation in modulating cell fate and survival. Desmosomal proteins may activate intracellular signaling through the modulation of expression levels and patterns, both of which can dramatically alter adhesion and cell proliferation [5,6]. In the interfollicular epidermis, Dsg2 is normally expressed at very low level and restricted to the proliferative basal cell layer. Recently, we developed a transgenic mouse model overexpressing desmoglein 2 (Dsg2) in the skin [5]. We determined that ectopic expression of Dsg2 activates multiple growth and survival pathways that may promote cancer development and progression. Although the Inv-Dsg2 transgenic mice developed precancerous papillomas and were more susceptible to chemically induced carcinogenesis, the mechanism by which Dsg2 induces these changes remains unclear. We recently showed that Dsg2 associates with caveolin-1 providing a mechanism for regulating mitogenic signaling and modulating the cell surface presentation, both of which may contribute to malignant transformation and tumor progression [7]. In this report, we sought to identify genes associated with the hyperproliferative phenotype by comparing the expression profile of Inv-Dsg2 transgenic mice with cDNA from wild-type mice as a control, via microarray analysis.

Specifically, we found Dsg2 was associated with the regulation of cystatin A (CSTA; mouse Csta1–3), also referred to as stefin A, acid cysteine protease inhibitor, keratolinin or “epidermal SH-protease inhibitor", a member of the Type 1 cysteine protease inhibitors [8–11]. CSTA is expressed primarily in epithelial and lymphoid tissues where it protects the proteolytic processing of cytoplasmic and cytoskeletal proteins by inhibiting cathepsins, the papain-like, lysozomal cysteine proteases [12–14]. It is therefore no surprise that CSTA possesses a number of biological functions, including a bacteriostatic role to protect tissues from cysteine proteases that are produced by invading pathogens [15]. In the skin, CSTA was originally identified in the cornified cell envelope and is suggested to play a role in barrier function targeting dust mite proteases [16]. More recently, we discovered that mutations in the *CSTA* gene are the underlying genetic cause of the skin fragility condition known as exfoliative ichthyosis with impaired cell-cell adhesion in the lower layers of the epidermis [17]. Additionally, recessive CSTA mutations can be associated with an acral peeling skin condition [18,19]. Here, we describe a number of studies investigating Dsg2 and CSTA in keratinocyte adhesion.

## Materials and Methods

### Ethics statement

All animal experiments were approved by the ethics committee that operates under Thomas Jefferson University Internal Animal Care and Use Committee (IACUC) approved protocols (642B and 642D).

### Generation of Inv-Dsg2 transgenic mice

We previously established transgenic mice expressing Dsg2 in the differentiating layers of the epidermis under the control of the involucrin (Inv) promoter (Inv-Dsg2) [5]. Briefly, the mouse *Dsg2*.*Flag* cDNA was subcloned into the pH3700-pL2 parental vector epitope at the *Not*I restriction site downstream of the involucrin promoter. Genotyping and characterization of the transgenic mice were previously described in detail [5]. Wild-type control and Inv-Dsg2 transgenic littermates of approximately 6 weeks old were used for these studies.

### Microarray analysis

Mouse skin tissues, stored in RNAlater (Qiagen, Valencia, CA), were pulverized with a mortal and pestle and homogenized in a Dounce homogenizer. Total RNA was isolated using TRIzol (Invitrogen, Carlsbad, CA) and RNeasy (Qiagen) according to the manufacturers’ protocols. Complementary DNA (cDNA) was synthesized from 2 μg RNA using hexa-random primers and M-MLV reverse transcriptase (SuperScriptIII System, Invitrogen). First strand cDNA were synthesized from 5 μg total RNA (2 wild-type and 2 transgenic samples) by reverse transcription and used to generate biotin-labeled cRNA by *in vitro* transcription. The microarrays were then processed using Streptavidin-Alexa 647 conjugate. After hybridization, washed slides were scanned to acquire fluorescent signals for each spot with a ScanArray XL-5000 confocal scanner (PerkinElmer, Boston, MA). To quantify the fluorescence intensities for each spot on the array, 16-bit images, were analyzed by Quant Array 3.0 software (PerkinElmer). After image processing, data were analyzed by GeneSpring 11.5 software (Agilent technologies, Santa Clara, CA). Background values based on signal intensities around each spot were subtracted, and results were normalized by quantile normalization and using baseline transformation median of all samples. Microarray data was submitted to Gene Expression Omnibus (Accession number: GSE62814).

Volcano plots were used to identify differentially expressed genes (greater than equal to 2 fold and statistical significance of p<0.05) using the Student’s *t*-test (unpaired) and no multiple testing corrections. The differentially expressed gene list was loaded into Ingenuity Pathway Analysis (IPA 9.0) software for network analysis.

### RT-PCR and quantitative real-time PCR (qRT-PCR) analysis of *Dsg2*, *Csta1*, *Csta2*, *Csta2l1*, and *Csta3*

cDNA was synthesized from 1 μg of RNA using the High Capacity cDNA Reverse Transcription Kit (Applied Biosystems, Foster City, CA). Exon-spanning qPCR primers were designed as follows: *Dsg2*, forward 5’-GAG GAA TTG AGT GCA GCA CAT AC-3’, reverse 5’-CTT GCT TCC ACC GTC AAG G; *Csta1*: forward 5’-TGC TAA CAA GGT CAG ACC TCA G-3’, reverse 5’-CCA TGG TTT TGT CAG TCT GGT-3’; *Csta2*: forward 5’- TGA ATG TAG GAC GTG GTT GC-3’, reverse 5’-GGG ATC AGG TCA AGT TGG AA-3’, *Csta2l1*: forward 5’-TGT CTT AAG TGG TAT TTC CAG TGA AAA CGA C-3’, reverse 5’- CAT CTC TTT ACA ATG GGG GGG GG-3’; *Csta3*: forward 5’- GCC CAT CAA TGA GTC AAG AAA-3’, reverse 5’-GGC CTC TGA AGA CTT TCA TGT G-3’ and for internal control *GAPDH*: forward 5’-CCC ATC ACC ATC TTC CAG GAG CGA-3’, reverse 5’-TCC ACC CTT CAA GTG GGC CCC-3’. cDNAs were amplified in PCR buffer containing 1 unit Taq DNA polymerase (Qiagen), 200 μM dNTPs, 1 unit Q solution, 0.5 μM of primers. Amplification of each of the cDNA was an initial denaturation step at 94^°^C for 3 min and 35 cycles of denaturation at 94^°^C for 30 sec, annealing at 58^°^C for 1 min and extension at 72^°^C for 1 min, followed by the last cycle of extension at 72^°^C for 7 min.

Gene expression levels were assayed by qRT-PCR analysis by using 1 μl of the cDNA and SsoFast EvaGreen Supermix (Bio-Rad Laboratories, Hercules, CA) according to a standard amplification protocol on an ABI Prism 7900 Sequence Detection System (Applied Biosystems). *GAPDH* expression was used to normalize data. The primer sequences employed for qRT-PCR for *Dsg2*, *Csta1*, *Csta2*, *Csta2l1*, and *Csta3* are included above.

### Immunohistochemistry and Immunoblotting

Unless otherwise indicated, all chemicals were from either Sigma (St. Louis, MO) or Fisher (Waltham, MA). For histology, skin tissues were fixed at room temperature overnight in a 10% formalin solution. Tissues were embedded in paraffin, sectioned (4 μm), mounted on glass slides, and stained with Hematoxylin and Eosin. For immunostaining, formalin-fixed paraffin-embedded tissues were heated to 60°C for 1 hr and deparaffinized in xylene (3 times), 100% EtOH (2 times), 95% EtOH (2 times), 75% EtOH, 50% EtOH, and H_2_O [20]. Antigens were retrieved by microwaving in Tris-EDTA Buffer (10 mM Tris Base, 1 mM EDTA solution, 0.05% Tween 20, pH 9.0). Tissues were then blocked in blocking buffer (5% normal goat serum, 1% BSA, 0.1% Ttriton X-100 in PBS) for 1 hr at room temperature (RT) then incubated with cystatin A antibody (1:50; AB4065, Millipore, Temecula, CA), Dsg2 Ab10 (1:10,000) and Flag M2 (1:500, Sigma) in blocking buffer, overnight at 4^°^C. Tissues were washed in PBS (2 times) and incubated with Alexa Fluor anti-Rabbit 488 IgG and anti-Mouse 594 IgG (1:400; Molecular Probes, Oregon) for 1 hr, at RT in the dark. Nuclei were stained with DAPI (1:1000, Sigma) for 1 min at RT in the dark followed by washing in PBS.

For Western blots, skin tissues were flash frozen in liquid nitrogen, pulverized with mortal and pestle and homogenized in RIPA buffer (50 mM Tris-HCl (pH 7.5), 150 mM NaCl, 1% Nonidet P-40, 0.5% deoxycholate, 0.1% SDS, protease inhibitor (PI) cocktail (Roche Diagnostics, Indianapolis, IN), 1 mM phenylmethylsulfonyl fluoride (PMSF) and phosphatase inhibitor cocktail (Fisher). Protein concentration was determined (Pierce BCA kit, Pierce Biotech, Rockford, IL) and immunoblotting was performed as described previously [21] with ~20 μg of protein in each lane resolved over 4–20% SDS-PAGE (Bio-Rad Laboratories, Hercules, CA). Primary antibodies used were: Flag M2 (1:2000, Sigma); Actin (1:5000; Calbiochem, San Diego, CA); Cystatin A (1:1000; Millipore, Temecula, CA); human Dsg2 (1:100; monoclonal Ab 10D2); DSP I/II (1:250; Clone 5–11F; [22]); vinculin (1:1000; AbCAM, Cambridge, UK). Signals were detected with the addition of secondary antibody HRP-conjugated (1:5000; Jackson Labs, Bar Harbor, ME), visualized by chemiluminescence (ECL; Amersham Biosciences, Piscataway, NJ). Alternatively, signals were detected using specific goat anti-mouse IR-Dye 800 CW (1:20,000; LI-COR Cat. 926–32210) or goat anti-rabbit IR Dye 800 CW (1:20,000; LI-COR Cat. 926–32211) and further analyzed and quantified by Odyssey IR imaging system (LI-COR Biosciences, Lincoln, NE). Western blots analyzed by chemiluminescence were quantified using ImageJ software developed at the National Institutes of Health (website: http://rsbweb.nig.gov/ij/). Statistical analysis was examined using Student’s *t* test with a *p* value of <0.05 considered significant (*p<0.05; **p<0.01; ***p<0.001), whereby quantified measurements from three independent experiments were normalized to Actin loading control.

### Cell culture and siRNA knockdown of *Dsg2* and *CSTA*

A431 (epidermoid carcinoma from American Type Culture Collection, Bethesda, MD) cells were maintained in DMEM supplemented with 10% FBS and 1% penicillin-streptomycin (P/S). A431 cells were grown to ~60% confluency on 6-well culture dishes, serum-starved for 4 hr with 0.5% FBS and 1% P/S prior to incubation with 100 nM scrambled siRNA control (D-001810–10, Dharmacon, Thermo Scientific, Lafayette, CO) or pooled siRNA to *Dsg2* (L-011645–00, Dharmacon) and *Csta* (L-010020–00, Dharmacon) using Lipofectamine RNAiMax (Invitrogen, Carlsbad, CA) and Opti-Mem medium (Invitrogen) according to the manufacturer’s protocol. Cells were returned to normal medium after 18 hr and incubated for 72 hr. Cells were lysed in 9 M Urea then prepared for Western blot analysis as described above. HaCaT cells were treated with siRNA by reverse transfection as per manufacturer’s instructions using Lipofectamine RNAiMAX. For immunofluorescence, cultured cells were fixed in MeOH -20°C for 10 min and permeabilized in 1% TX-100 for 5 min. After non-specific sites were blocked, cells were incubated with antibody 10D2 for Dsg2 or CSTA antibody (see above).

In some experiments, cells treated with scrRNA or *CSTA* siRNA were seeded on BioFlex 6-well plates containing a flexible rubber membrane coated with pronectin (Flexplates, Flexcell International, Hillsborough, NC, USA). Cells were grown to ~80% confluency, and the monolayers were stressed mechanically with a Flexcell FX-4000 Tension System (Flexcell International, Hillsborough, NC, USA) as previously described in detail [17] (Blaydon et al., 2011). Cells were stretched for 0–4 hr, and then fixed and stained for Dsg2 (1:500; Rabbit Ab10; [23]) and keratin 14 (1:100; Clone LL001; Santa Cruz Biotechnology, Inc.). Cells were also lysed in Laemmli buffer for Western blotting.

### Knockdown of Dsg2 by shRNA

A431 cells stably transfected with the pSuper Retro-puro vector carrying specific shRNA targeting nucleotides to GFP (shGFP) or Dsg2 (shDsg2) were established as described previously in detail [24]. Briefly, two oligos (‘5-GAT CCC CGA GAG GAT CTG TCC AAG AAT TCA AGA GAT TCT TGG ACA GAT CCT CTC TTT TT-3’ and ‘5-AGC TTA AAA AGA GAG GAT CTG TCC AAG AAT CTC TTG AAT TCT TGG ACA GAT CCT CTC GGG-3’) were synthesized, annealed, and ligated into pSuper Retro-puro (Oligo-Engine, Seattle, WA). Retrovirus production and infection was performed in Phoenix cells. Transfected A431 cells were maintained in DMEM supplemented with 10% FBS and puromycin (2 μg/ml).

### Dispase-based cell dissociation assay

To assess the effects of cystatin A and Dsg2 on cell adhesion, Mock and shDsg2 A431 cells were plated on six-well culture dishes at a density of 2 X 10^5^ cells/well and then treated with 50 nM scrambled siRNA control or siRNA to *Csta* as described above. After 72 hr, cells were washed with Hank’s Balanced Salt Solution and incubated with dispase (BD Biosciences, San Diego, CA, USA) for 5 min. The lifted cell sheets were subjected to dispase-based dissociation assay by pipetting five times by using a 1-ml pipette. Cell fragments were fixed in 3% formalin solution and stained with crystal violet. Experiments were repeated at least 3 times. We note here, that these experiments were performed without adding extra calcium.

## Results

### Microarray analysis comparing wild-type and Inv-Dsg2 transgenic mouse skin

We recently showed that overexpression of Dsg2 under the control of the involucrin promoter in the skin of transgenic mice (Inv-Dsg2) resulted in hyperplasia of the epidermis and enhanced sensitivity to tumor induction [5]. Moreover, Inv-Dsg2 transgenic mice also developed benign papillomas and were more susceptible to chemically induced two-stage skin carcinogenesis. These findings prompted us to perform a comparison of the gene expression profiles between the Inv-Dsg2 transgenic and control mice. To avoid background fluctuations and variations, our Dsg2 transgenic mice were crossed back at least 5 generations to C57BL6 background. We opted to use full-thickness skin in order to observe any changes in gene expression in the dermis that may have been introduced by the overlying epidermis. Thus, back skin from littermates at 6 weeks after birth was used.

H&E-stained tissue sections of transgenic skin samples exhibited extensive hyperplasia compared to that of wild-type skin (S1A Fig.). Expression of the transgene was confirmed by Western blot analysis whereby the Flag antibody detected the 160 kDa Dsg2.Flag protein in the transgenic but not in the wild-type mouse skin (S1B Fig.). In addition, immunofluoresence microscopy verified the expression of the Flag-tagged Dsg2 protein in the superficial epidermis of the transgenic, but not wild-type mice (S1C Fig.). These results demonstrate, as previously described [5], that ectopic expression of Dsg2 induces epidermal hyperplasia without altering the endogenous expression of Dsg2. In both mouse and human interfollicular epidermis, Dsg2 is expressed at significantly low level in the basal cell layer, and that expression level decreases further with development [5,25]. In adult tissues, only a minute signal is detected when the exposure level is enhanced. Attempts were made to co-immunostain for Dsg2 and Csta using anti-Dsg2 antibodies. However, due to the nature of those antibodies, low expression of endogenous Dsg2, and the background being too high (data not shown), we opted to use anti-Flag antibodies to detect the Flag-tagged Dsg2 transgene. Dsg2-Flag is expressed in the superficial epidermis under the control of the involucrin promoter of the transgenic mice as previously described in detail [5].

**Fig 1:**
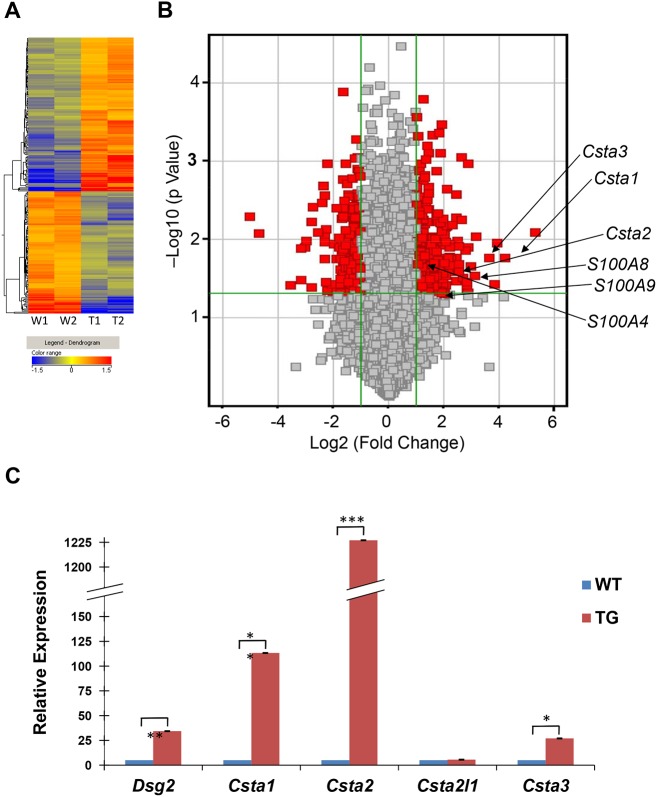
Differential gene expression between Inv-Dsg2 transgenic and wild-type mouse skin. (A) Total RNA was isolated from the skin of 2 wild-type and 2 Inv-Dsg2 transgenic mice, reverse transcribed, biotin-labeled and applied to a mouse cDNA microarray. The dendogram (heat map) shows that 492 genes were either up-regulated (red/orange) or down-regulated (blue/green) in transgenic (T1 and T2) and control (W1 and W2) mice. (B) Volcano plot shows the log2 (fold change) in x-axis versus the—log10 (p value) in the y-axis. The points having a fold-change less than 2 (log2 = 1) are shown in gray. The vertical green lines demarcate where the fold change equals 2 (right line) or equals—2 (left line). The horizontal green line demarcates where the p value is 0.05, with points above the line having p<0.05 and points below the line having p>0.05. Depicted in red are the genes that exhibit a greater than 2 fold change with a p>0.05 in transgenic epidermis as compared to control. The arrows indicate genes of interest. (C) Quantitative real-time RT-PCR analysis reveals an average of 34.33±1.32 fold increase in Dsg2 RNA expression in Inv-Dsg2 transgenic (Tg) compared to that of wild-type (WT). In addition, RNA expression for transgenic relative to control were: *Csta1*, 113.24 ±2.23; *Csta2*, 1227.04 ±1.26; *Csta2l1*, 1.11 ±0.47; *Csta3*, 26.97±0.44 (Bar = mean ± s.d.; (*p< 0.05; **p<0.01; ***p<0.001; Student’s *t* test).

Total RNA samples isolated from the skin of two transgenic and two wild-type mice were used to generate biotin-labeled cRNA probes and then hybridized to oligonucleotide microarrays representing 21,619 mouse genes (Fig. 1A). A comparison of the average gene signals revealed approximately 492 genes altered by more than two fold in response to Dsg2 expression (Table 1 and S1 Table). A volcano plot revealed that 275 transcripts were upregulated and 217 downregulated with statistical significance (p<0.05, shown in red, Fig. 1B). These 492 genes modulated in response to Dsg2 were further analyzed by the Ingenuity Analysis Program (Qiagen), which describes the top 5 gene networks according to their degree of relevance to the Network Eligible Molecules in our dataset (S2 Table). This analysis revealed strong association with the Cell Cycle (S2A Fig.) and Cancer Regulation pathways (S2B Fig.). A significant number of genes involved in different phases of cell cycle regulation were upregulated (red) in response to Dsg2 (and further summarized in S3 Table). Similarly, many genes involved in tumorigenesis were also upregulated in response to Dsg2 including, the mitotic checkpoint protein kinase (*BUB1*) (S1 Table) and the distal-less 4-homeobox (*DLX4*) and the cancer susceptibility protein (*BRCA1*) (S2B Fig.). Interestingly however, two members of the forkhead family of transcription factors, *FOXC1* (-9.0 fold) and *FOXC2* (-11.7 fold), were downregulated in the Inv-Dsg2 transgenic mice. FOXC2, in particular, induces epithelial-mesenchymal transition and plays a role in eyelid closure [26,27]. Knockdown of FOXC2 expression in breast cancer cells abrogates their metastatic potential [28], implicating its role in cancer development. The role of FOXC2 in the skin homeostasis and cancer development is not known.

**Table 1:** Differentially expressed genes in response to Dsg2.

Fold	Gene	Mapped	Un-mapped	Up	Down
> 2.0	492	468	24	275	217
> 2.5	247	234	13	144	103
> 3.0	152	144	8	54	98

Table 2 lists the top ten genes that were modulated in response to Dsg2. This list comprised of encoding proteins implicated in tumorigenesis including L-threonine dehydrogenase (*TDH*, 39.2 fold) and cysteine-rich secretory protein (*CRISP3*, 8.8 fold). The most surprising finding was the enhanced expression of CSTA (*Csta1*, 18.4 fold; *Csta2*, 5.7 fold; and *Csta3*, 12.4 fold) and several members of the S100 family of calcium binding proteins (*S100A8*, 7.9 fold; *S100A9*, 4.1 fold; and *S100A4*, 2.3 fold) (Fig. 1B; Table 3). The S100A8 and S100A9 proteins form a complex known as calprotectin, which exhibits antimicrobial activities and is shown to protect epithelial cells against invading pathogens [29]. Furthermore, the gene expression of four members of the cyclin family of proteins were also increased, including cyclin A2 (*Ccna2*, 5.8 fold), cyclin B2 (*Ccnb2*, 5.4 fold), cyclin B1 (*Ccnb1*, 4.6 fold), and cyclin E1 (*Ccne1*, 2.6 fold), whilst expression of one member, cyclin A1 (*Ccna1*, -2.0 fold) was found to be decreased (Table 3). The cyclin family of proteins regulates cyclin-dependent kinases and controls the progression of cells through the cell cycle [30]. This finding is consistent with our previous results showing that Dsg2 enhances cell proliferation. CSTA has been shown to possess anti-apoptotic activity [31], is upregulated in several epithelial-derived malignancies including SCC [32], and is mutated in inherited cell-cell adhesion defective epidermal disorders [17–19]. Furthermore, CSTA inhibits cathepsins [33,34], which are also deregulated in skin cancer [35–37]. For those reasons, we focused the remainder of this study on CSTA. The functional relevance of the S100 and cyclin proteins will be examined in detail in a future study.

**Table 2:** Top ten genes up- or down-regulated in response to Dsg2.

GenBank Accession #	Gene	Gene Product	Fold	p-value	Functions
**Top ten genes up-regulated**					
NM_021480	TDH	L-threonine dehydrogenase	39.18	8.1E-03	Enzyme
M92417	Csta1/StfA1	Cystatin A1/Stefin A1	18.39	1.7E-02	Cysteine proteinase inhibitor
D45850	AKR1C4	Aldo-keto reductase family 1 member C4	15.18	1.1E-02	Enzyme
AK007978	Unknown	Unidentified EST	14.06	3.7E-02	Unknown
M92419	Csta3/StfA3	Cystatin A3/Stefin A3	12.37	1.7E-02	Cysteine proteinase inhibitor
AJ251685	GPNMB	Glycoprotein (transmembrane) nmb	9.09	9.1E-03	Pigmentation
NM_009638	CRISP3	Cysteine-rich secretory protein 3	8.79	2.9E-02	Sperm regulation
M83218	S100A8	S100 calcium binding protein A8	7.86	2.2E-02	Casein kinase inhibitor
AK010010	Unknown	Unidentified EST	7.40	4.8E-02	Unknown
AJ237585	Ncapg	Non-SMC condensin I complex, subunit G	7.39	1.3E-02	**Chromatin condensation**
**Top ten genes down-regulated**					
NM_008791	*PCP4*	Purkinje cell protein 4	-6.58	3.9E-03	Calmodulin-mediated signaling
AK019744	Unknown	Unidentified EST	-6.84	6.1E-03	Unknown
AF070470	*SMOC1*	SPARC related modular calcium binding 1	-6.89	4.1E-02	Matrix assembly and cell adhesiveness
NM_007568	*BTC*	Betacellulin	-7.75	1.0E-02	EGF receptor ligand
AK018865	Unknown	Unidentified EST	-8.22	1.3E-02	
NM_008592	*FOXC1*	Forkhead box C1	-8.95	3.3E-02	Transcription regulation
BC014714	*HMGCS2*	3-Hydroxy-3-methylglutaryl-CoA synthase 2	-8.98	1.3E-02	Enzyme
NM_013519	*FOXC2*	Forkhead box C2	-11.69	3.8E-02	Transcription regulation
AK004289	Unknown	Unidentified EST	-25.73	8.3E-03	Unknown
AK009582	*PBRM1*	Protein polybromo 1	-32.82	5.1E-03	Chromatin-remodeling

**Table 3:** Upregulation of cystatin A, S100 and cyclin genes in Dsg2 transgenic mice.

Gene symbol	Name	Fold	p-value
*Csta1/Stfa1*	Cystatin A1 (Stefin A1)	18.39	1.7E-02
*Csta3/Stfa3*	Cystatin A3 (Stefin A3)	12.37	1.7E-02
*Csta2/Stfa2*	Cystatin A2 (Stefin A2)	5.71	2.6E-02
*S100A8*	S100 calcium binding protein A8	7.86	2.2E-02
*S100A9*	S100 calcium binding protein A9	4.08	5.0E-02
*S100A4*	S100 calcium binding protein A4	2.28	2.2E-02
*Ccna2*	Cyclin A2	5.8	2.1E-02
*Ccnb2*	Cyclin B2	5.4	1.7E-02
*Ccnb1*	Cyclin B1	4.6	1.8E-02
*Ccne1*	Cyclin E1	2.6	3.3E-03
*Ccna1*	Cyclin A1	-2.0	2.9E-02

### Confirmation of Dsg2-modulation of CSTA expression

The microarray results were confirmed by examining the expression of mouse Csta mRNA (1, 2, 2L1 and 3) by RT-PCR (S3 Fig.) and real-time qPCR (Fig. 1C). First, total RNA was isolated from the skin of two individual representative wild-type and Inv-Dsg2 transgenic mice, cDNA was generated and used as a template for PCR confirming the upregulation of *Dsg2*, *Csta1*, *Csta2*, and *Csta3* in transgenic as compared to control mice (S3 Fig.). No change in RNA expression was observed with *Csta2l1* and *GAPDH*. Csta2l1 is highly expressed in the fetal liver, bone marrow, and spleen and was used here as a negative control for skin [38]. The RT-PCR data was further validated by real-time qPCR using skin biopsies from 3 wild-type and 3 transgenic mice (Fig. 1C). Again, up-regulation of *Dsg2* (34.33±1.32) was observed in skin from transgenic mice as compared to skin from control animals. By real-time qPCR, we observed an increase in *Csta1* (113.24±2.23), *Csta2* (1227.04±1.39), and *Csta3* (26.97±0.44), but not *Csta2l1* (1.11±0.47). With the exception of *Csta2l1*, all changes from transgenic compared to control samples were statistically significant. Interestingly, the fold change difference obtained by real-time qPCR was significantly different than obtained from microarray analysis. This is not unusual and has been observed in other systems [39]. In summary, qPCR analysis confirmed the microarray data demonstrating that ectopic expression of Dsg2 in the epidermis caused an increase in the RNA expression of the mouse *Csta* family of proteins and that this enhanced expression is regulated by the overexpression of Dsg2 in the skin.

**Fig 2:**
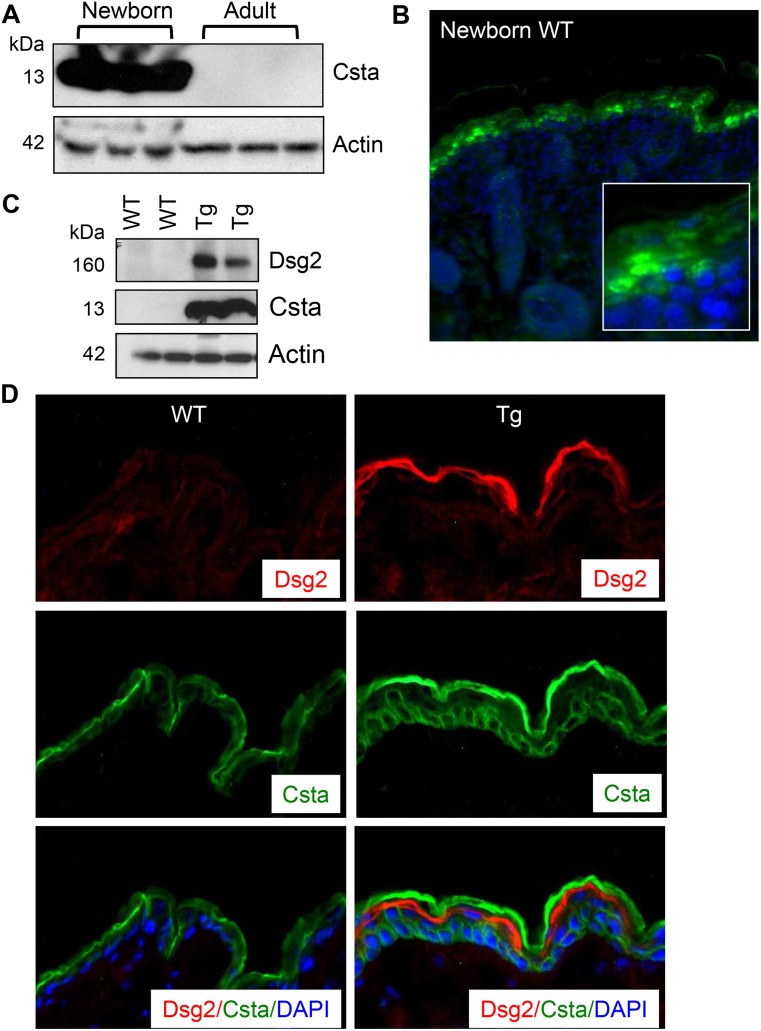
Dsg2 enhances cystatin A expression *in vivo*. (A) Western blot analysis of skin lysates from 3 newborn and 3 adult C57Bl6 mice shows high expression of Csta in newborn but virtually undetectable in adult skin. Actin was used as a control for equal loading. (B) Immunofluorescent staining confirms the Western blotting results showing high level of Csta in newborn wild-type mouse skin. Enlarged image in inset shows cytoplasmic as well as nuclear staining for Csta. (C) Western analysis for Dsg2 and Csta in adult wild-type and Inv-Dsg2 transgenic mouse skin. The results showed expression of the Flag-tagged Dsg2 and Csta in the transgenic but not wild-type mice. Actin showed equal loading. (D) Immunofluorescence was performed on adult skin of wild-type and transgenic mice revealing increased levels CSTA in transgenic skin. Nuclei were counter-stained with DAPI (blue).

Next, skin lysates from newborn and adult control mice (n = 3 each) were immunoblotted for Csta revealing high level in newborn skin, but dramatically decreased in adult skin (Fig. 2A), corroborating with previous observations [40]. Interestingly, we observed both cytoplasmic and nuclear staining for Csta in newborn mouse skin by immunofluorescence (Fig. 2B). No significant difference in Csta level was observed between the wild-type and Inv-Dsg2 transgenic newborn mice (not shown). However, since Csta was low in the skin of adult mice, ectopic expression of the Dsg2 dramatically enhanced Csta level (Fig. 2C). The Flag antibody detected the Flag-tagged Dsg2 protein in the transgenic but not the wild-type mice (Fig. 2C). Similar results were observed using the Dsg2 specific antibody 10D2 demonstrating that ectopic expression of Dsg2 in the superficial epidermis did not alter the endogenous Dsg2 level (data not shown). Due to the nature of the antibodies, we were unable to perform co-immunostaining for Dsg2 and Csta. However, conditions were optimized to immunostain for the Flag-tagged Dsg2 (anti-Flag antibodies) and Csta (Fig. 2D). In the Dsg2 Tg skin, Csta was expressed in cells regardless of transgene expression, thus suggesting a non-autonomous effect. Staining for Csta was also detected in the corneocytes of wild-type mice and increased in the transgenic mice (Fig. 2D). Although Csta was not detected by Western blot using total skin lysates, we cannot rule out that in the cornified envelope, Csta may be highly cross-linked and thus resistant to lysis in SDS/DTT lysis buffer.

Unlike in human with only one *CSTA* gene, there are multiple *Csta* genes in mice [41]. While the microarray analysis detected three *Csta1*, *Csta2* and *Csta3*, it is unknown which isoform is expressed in the mouse skin. Furthermore, it is not known which mouse Csta is recognized by the commercially available antibodies since the epitopes for these have not been mapped. However, we speculate that the antibodies may recognize all Cstas due to high sequence homology between human and mouse and between the different mouse isoforms.

### Dsg2 modulates CSTA expression

**Fig 3:**
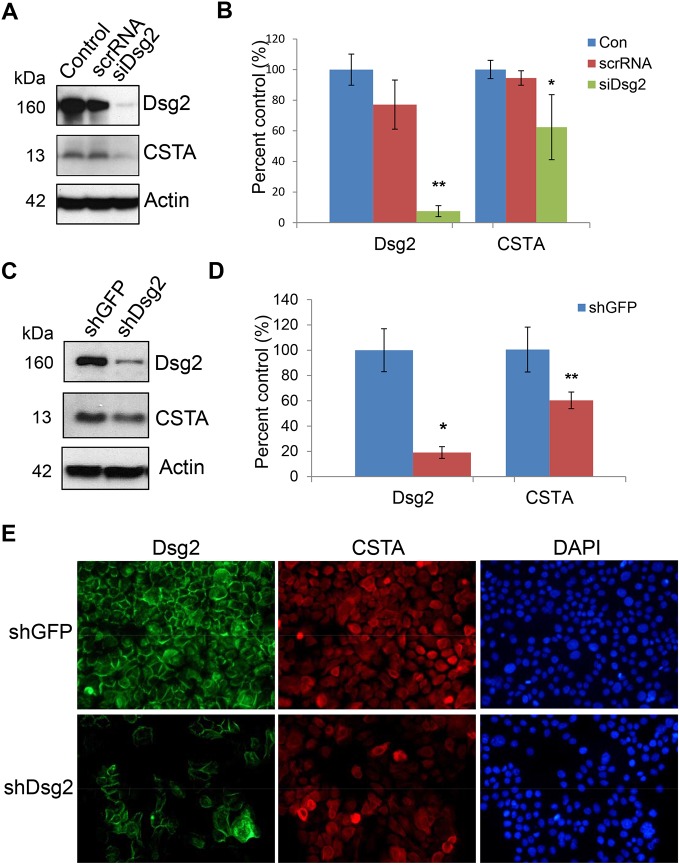
Modulation of CSTA expression by Dsg2. (A) A431 cells were treated for 72 hr with 100 nM of scrambled RNA or *Dsg2* siRNA. Western blot analysis for Dsg2 and cystatin A shows that knockdown of Dsg2 reduced CSTA level. Immunoblotting for Actin showed equal loading. (B) The Western blot results were quantified and expression level of each band was normalized against Actin. Values shown are percentage of expression against control untreated. The results showed significant reduction in Dsg2 in response to *Dsg2* siRNA but not scrambled siRNA, while knockdown of Dsg2 slightly reduced the expression of CSTA. The change was statistically significant. Bar = mean ± s.e.m. *p<0.05 and ***p<0.001 using Student’s *t* test. (C) A431 cells were stably transfected with shRNA to GFP (shGFP) or Dsg2 (shDsg2) and selected in puromycin. Immunoblotting showed loss of Dsg2 reduced CSTA expression in the shDsg2 cells, as compared to the shGFP cells. Actin was used as a loading control. (D) Quantification of the Western blot results showed reduction in Dsg2 in the shDsg2 cell as compared to the shGFP cells and knockdown of Dsg2 reduced CSTA expression. Bar = mean ± s.e.m. *p<0.05 and ***p<0.001 using Student’s *t* test. E) Immunofluorescence of Dsg2 and CSTA showed that knockdown of Dsg2 reduced the expression of Dsg2 and CSTA in the A431-shDsg2 as compared to A431-shGFP cells. Nuclei were counter-stained with DAPI (blue).

To determine if Dsg2 modulates human CSTA in keratinocytes, A431 cells were treated with siRNA specific for Dsg2 (siDsg2), CSTA (siCSTA) or scrambled RNA (scrRNA). Three days post-transfection, with scrRNA and siCSTA, Western blot analysis showed that knockdown of CSTA did not affect the expression of Dsg2 (S4 Fig.). In contrast, a dramatic reduction of Dsg2 by siDsg2 but not scrRNA, resulted in small but reproducible reduction of CSTA (Fig. 3A,B). To further confirm the effect of Dsg2 on CSTA expression, stable A431 cell lines with knockdown of Dsg2 using short hairpin RNA (shRNA) were established [24]. Immunoblotting revealed that Dsg2 was significantly down regulated in shDsg2 cells, as compared to shGFP cells (Fig. 3C,D). Similar to the results observed using siRNA, knockdown of Dsg2 by shRNA slightly suppressed the expression of CSTA (Fig. 3C,D). A431-shGFP and A431-shDsg2 were immunostained for Dsg2 and CSTA showing knockdown of Dsg2 in the A431-shDsg2 cells resulted in reduction of CSTA (Fig. 3E). In summary, these results suggest that CSTA is modulated in part by Dsg2.

### Effect of CSTA on cell-cell adhesion

**Fig 4:**
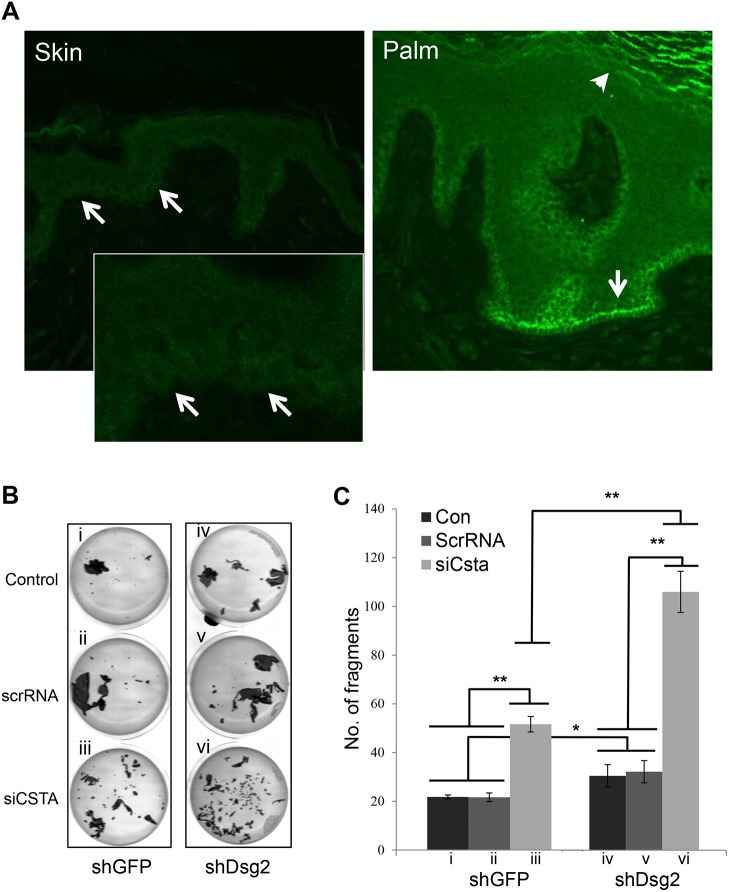
Modulation of cell adhesion by CSTA and Dsg2. (A) Immunofluorescence of normal human skin (A) and palm (B) showing low levels of Dsg2 in the basal layer (arrows) of the normal skin (inset: enlarged image) but high levels in both the basal and differentiated layers (arrow head) in the palm. Note: Immunostaining was performed at the same time and images were captured at the same exposure. (B) A431-shGFP and A431-shDsg2 cells were treated with scrambled RNA or siRNA to *CSTA* for 72 hr and then subjected to the *in vitro* mechanical stress dispase-based dissociation assay. Bright field images showing loss of Dsg2 or CSTA induced fragmentation and the loss of both had a synergistic effect on cell adhesion. (C) Graph showing the number of fragments for each condition. i, shGFP; ii, shGFP + scrRNA; iii, shGFP + siCSTA; iv, shDsg2; v, shDsg2 + scrRNA; vi, shDsg2 + siCSTA. Bar = mean ± s.e.m. *p< 0.05 and **p<0.01 using Student’s *t* test.

In the autosomal recessive disorder exfoliative ichthyosis, loss-of-function mutations in *CSTA* results in coarse peeling of the skin on the palms and soles and detachment occurring in the lower epidermis with abnormal desmosomes [17]. Here, we wanted to assess whether Dsg2 plays a role exfoliative ichthyosis by synergizing with CSTA to modulate cell adhesion. Normal human back skin and palm were immunostained for Dsg2 showing low levels in the basal layer of the interfollicular epidermis but high levels in the both the basal and superficial layers in palmoplantar epidermis (Fig. 4A). These results are similar to our previous findings [23].

Next, the impact of Dsg2 and CSTA on cell-cell adhesion was assessed. We attempted multiple times to express CSTA in keratinocytes using different expression plasmids (His or HA tag) but were unsuccessful possibly due to CSTA being negatively regulated by the Ras/Raf-1/MEK1/ERK pathway [42]. Hence, we opted to knockdown CSTA. A431-shGFP and A431-shDsg2 cells were subjected to the in vitro dispase-based keratinocyte dissociation assay 72 hr post-treatment with scrRNA or siCSTA to knockdown CSTA. Sheets of cells were lifted from the culture dish using dispase and disrupted by pipetting. Cell fragments were photographed and counted. Independent knockdown of either Dsg2 (Fig. 4Aiv, Biv) or CSTA (Fig. 4Aiii, Biii) resulted in an increase in fragmentation when compared to untreated cells (Fig. 4Ai, Bi) or scrRNA treated cells (Fig. 4Aii, Bii). In addition, the dual loss of Dsg2 and CSTA (Fig. 4Avi, Bvi) further enhanced fragmentation.

**Fig 5:**
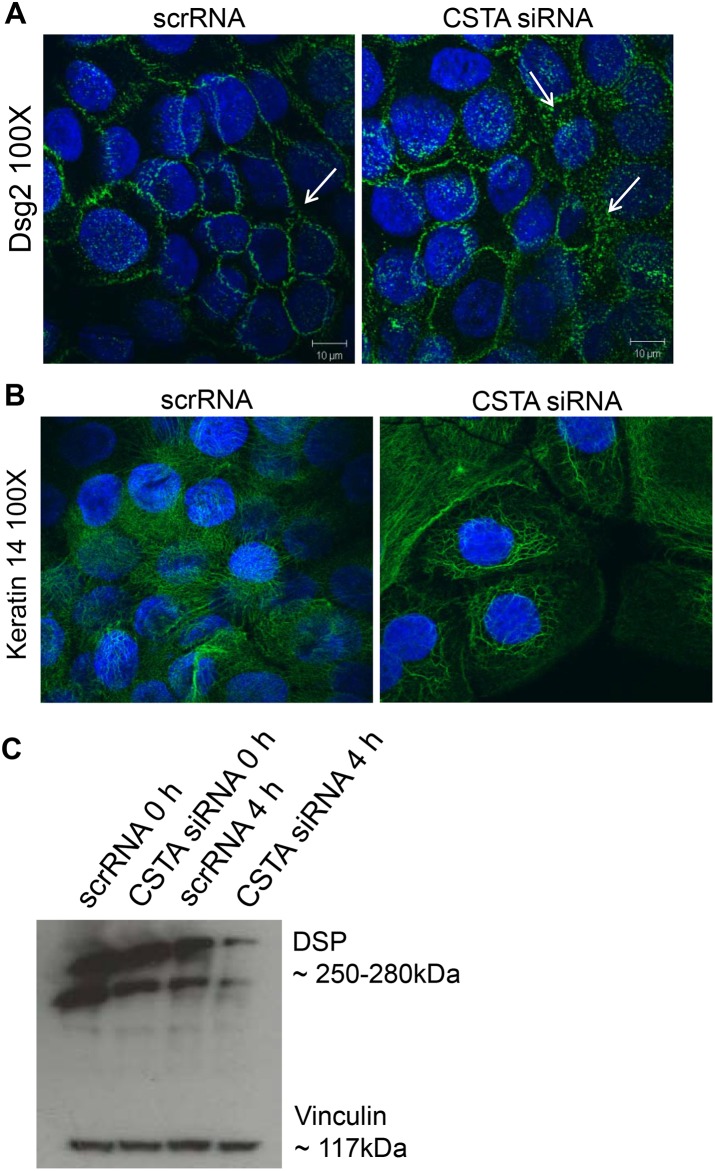
Loss of CSTA leads to destabilized intercellular connections. Cells were treated with non-targeting pool scrRNA or with *CSTA* siRNA (CSTA KD) followed by mechanical stretching for 4 hr. Cells were allowed to adhere, fixed, and immunostained for Dsg2 (A) and cytokeratin 14 (B) or lysed in Laemmli buffer and immunoblotted for desmoplakin (C). Knockdown of CSTA in keratinocytes resulted in cytoplasmic relocalization of Dsg2, breakage of cytokeratin intercellular connections, and loss of the desmosomal protein, desmoplakin.

Finally, to demonstrate that loss of CSTA leads to decreased cell-cell adhesion by disrupting desmosomal structures, we treated HaCaT keratinocytes with scrRNA or CSTA siRNA, subjected to 4 hr mechanical stretching and then immunostained for Dsg2 (Fig. 5A) and keratin 14 (Fig. 5B). CSTA knockdown led to increase in cytoplasmic localization of Dsg2 and retraction of cytoskeletal organization. Furthermore, Western blotting showed a decrease in desmoplakin levels in CSTA knockdown stretched monolayers compared to control cells stretched for the same number of hours (Fig. 5C). The changes observed in components of the desmosome suggest that the cell-cell breakage occurs at the desmosomes. In summary, CSTA plays a role in epithelial cell adhesion as loss of CSTA rendered the cells susceptible to mechanical disruption by destabilizing the desmosomal structures.

## Discussion

Dsg2 is up-regulated in many epithelial-derived tumors such as basal and squamous cell carcinomas [23]. In this report we showed that ectopic expression of Dsg2 in the murine epidermis dramatically altered the expression of many genes involved in cell cycle regulation and cancer development, including the expression of the cysteine protease inhibitor, CSTA. This study demonstrates for the first time that a desmosomal cadherin can modulate the expression of CSTA both *in vivo* and *in vitro*.

CSTA was originally identified as a precursor protein of the cornified cell envelope [43] and reduced expression of CSTA contributes to a defective epidermal barrier in the skin condition known as atopic dermatitis [44,45,16]. However, *CSTA* message has been localized to the lower epidermis [46]. Here we show that Csta, similar to Dsg2, was expressed at high level throughout the newborn epidermis but decreased during development. In the epidermis of adult mice, minor signal was detected in the differentiated layers [20]. Ectopic expression of Dsg2 in transgenic mice enhanced Csta expression suggesting perhaps that Csta may be regulated in part by Dsg2 [5]. Concomitant with the expression level of Dsg2 and Csta, the skin of newborn mice is hyperplastic (~5–6 layers) as compared to the 2–3 layers in adult mice. An increase in Dsg2 with upregulation of Csta in the Inv-Dsg2 transgenic mice also induced hyperplasia [5]. In human keratinocytes, CSTA displays anti-apoptotic activity through its inhibition of UVB-induced caspase 3 activation, thereby suppressing UVB-induced apoptosis suggesting that CSTA may play an important role in controlling cell growth, differentiation and survival [44].

Our results complement the recent findings showing that loss-of-function mutations in the *CSTA* gene is the underlying cause of the skin condition exfoliative ichthyosis [17]. The skin of affected individuals displayed hyperkeratosis and superficial exfoliation. Interestingly however, loss of CSTA did not affect barrier function or terminal differentiation. Instead, histology of the exfoliative ichthyosis palmoplantar skin exhibited loss of cell-cell adhesion in the deep epidermis. Particularly, the basal and immediate suprabasal layers showed signs of disrupted epidermal structure and disorganization of desmosomes. Here, we have shown that loss of CSTA and Dsg2 enhanced cell-cell disadhesion (Fig. 4B,C) and that CSTA modulated desmosome stability (Fig. 5). These results suggest that Dsg2 may play a role in the skin fragility phenotype of exfoliative ichthyosis as it is highly expressed in both the basal and superficial layers in palmoplantar tissues (Fig. 4A). We note here that we cannot rule out the role of other desmosomal proteins, such as desmoglein 3 and desmocollins 2 and 3, that are also expressed in the deep epidermis and that may affect the expression and/or function of CSTA [20,47]. The crosstalk between these other cadherins and CSTA will be the subject of future studies.

The impact of the Dsg2-CSTA influence on cell adhesion may play a yet unrecognized role in the skin fragility condition pemphigus. Pemphigus is a group of autoimmune skin blistering diseases caused by loss of cell-cell adhesion due to autoantibodies binding to Dsg1 and Dsg3 resulting in the internalization of desmosomes [48]. Passive transfer of pathogenic pemphigus IgG into neonatal mice produces epidermal blister formation similar to those observed in patients [49,50]. Our previous study showed that compared to wild-type mice, ectopic expression of Dsg2 in the superficial epidermis rendered the Inv-Dsg2 transgenic mice more resistant to blister formation by pemphigus foliaceus IgG [51]. Here, we show that loss of Dsg2 disrupted cell-cell adhesion and this effect was further amplified with loss of CSTA. Thus, we speculate that in the Inv-Dsg2 transgenic mice, forced expression of Dsg2 increased the CSTA level thereby enhancing cell-cell adhesion and possibly protecting the epidermis from acantholysis-associated PF blister formation. Dsg2 is often upregulated in the skin of affected patients [48] and keratinocytes from Dsg3 knockout mice upregulate Dsg2 [52]. These findings support the notion that induction of Dsg2 in lesional skin of pemphigus patients could be a compensatory mechanism to enhance cell-cell adhesion and thus protect the patients from blister formation. It would be interesting to assess the expression of CSTA in pemphigus patients’ skin.

Although evidence supports a causal role for proteases in malignant progression of human cancers, the role of CSTA is somewhat complicated and controversial [53]. In head and neck SCC, expression of CSTA has been reported to be down-regulated in some patients while up-regulated in others [54,55]. Overexpression of CSTA has been detected in a variety of human cancers including lung, breast, head and neck, vulva, cervix, esophagus and prostate, and in some mouse sarcomas. In some forms of highly malignant and metastasizing breast cancer, there is a correlation between increased CSTA expression and poor prognosis [56]. Upregulation of CSTA is also detected in another transgenic mouse model overexpressing the early gene region of the human papillomavirus type 8, these mice develop papillomas similar to our Inv-Dsg2 mice [57]. The role of Dsg2 in cancers is equally controversial and the expression level is dependent on the tumor type. We, and others have shown that while Dsg2 expression in the interfollicular epidermis is demonstrably low [20], it is markedly increased in skin, prostate, and colon cancers [23, 58–61]. Interestingly, in diffuse-type gastric cancers, decreased expression of Dsg2 is associated with poor prognosis suggesting a complex role for Dsg2 in oncogenesis, serving as a tumor enhancer or suppressor [62].

The mechanism by which Dsg2 modulates CSTA expression remains to be determined and future studies to assess the Dsg2-DNA interactions and map the Dsg2 binding sites are necessary. However, we recently demonstrated that Dsg2 might have an impact on signaling by binding to the scaffolding protein caveolin-1, the major components of lipid rafts, caveolae [7]. Caveolins and caveolae have been implicated as regulators of key cellular functions by modulating mitogenic signaling pathways such as Wnt/β-catenin/Lef-1 [63]. Thus, Dsg2 may enhance the PI 3-kinase/AKT, MEK-MAPK, STAT3 and NF-kappaB signaling pathways resulting in altered CSTA gene expression. The up-regulation of CSTA may reflect a compensatory mechanism to offset the increase in activity of proteases such as cathepsin that modulate matrix remodeling during disease progression. Alternatively, an increase in CSTA activity in tumors may counter the activation of apoptosis induced by the tumor necrosis factor alpha and cathepsin B [64]. Thus, whether CSTA is an oncogene or a tumor suppressor is unresolved. However, the general consensus is that in many cancers, there is an imbalance between the proteases and their respective cystatin inhibitors [65–67].

## Conclusions

In this report, we demonstrated that Dsg2 plays an active role in modulating epithelial cell growth and survival by altering the epithelial gene transcriptome and modulating the expression of proteins that are markers or prognostic indicators of the dysplastic phenotype including, CSTA.

## Supporting Information

S1 Fig Upregulation of Dsg2 in the Inv-Dsg2 transgenic mice. (A) H&E-staining shows epidermal hyperplasia in the Inv-Dsg2 transgenic skin compared to wild-type control. (B) Western blot analysis of Flag shows the Flag-tagged Dsg2 in the transgenic but not wild-type skin. Immunoblot with anti-Actin antibody served as loading control for protein lysates. (C) Immunofluorescent analysis reveals expression of Dsg2 in the differentiated layers of the transgenic epidermis. Nuclei were stained with DAPI (blue). Scale bar, 200 μm. Tg, transgenic; WT, wild-type.
(TIF)

S2 Fig Functional gene networks identified using Ingenuity Pathway Analysis (IPA) software from differentially expressed genes between wild-type and Inv-Dsg2 transgenic mice. The IPA analysis revealed the top functional gene networks to be cell-cycle (A) and cancer (B) composed of multiple genes, many of which are involved in skin cancers (Cyclins, S100 family, FOXC1/2 and BRCA1 genes) to be most differentially expressed by Dsg2 compared to wild-type. Nodes represent genes and their level of color intensity is related to its level of expression (*red*, up-regulation; *green*, down-regulation). Uncolored nodes means these genes were not identified as differentially expressed and were integrated as part of the network analysis based on the information in the IPA databases.
(TIF)

S3 Fig RT-PCR analysis of *Dsg2* and *Csta* expression. RT-PCR showed that mRNA expression of *Dsg2*, *Csta1*, *Csta2* and *Csta3* were relatively higher in the Inv-Dsg2 transgenic skin compared to that of wild-type. *Csta2l1* expression was used as a control.
(TIF)

S4 Fig Effect of *CSTA* knockdown on Dsg2. A431 cells were treated for 72 hr with 100 nM of scrambled RNA or *CSTA* siRNA and total protein lysate was immunoblotted for Dsg2 showing that knockdown of CSTA had no effect on Dsg2 expression.
(TIF)

S1 Table Dsg2-dependent Gene Changes. (PDF)

S2 Table Associated Network Functions. (PDF)

S3 Table Changes in Expression of Cell Cycle Genes in Response to Dsg2. (PDF)
