# Supplementary figures and images for: Cell Cycle- and Cancer-Associated Gene Networks Activated by Dsg2: Evidence of Cystatin A Deregulation and a Potential Role in Cell-Cell Adhesion
Source: PLoS One. 2015 Mar 18;10(3):e0120091. doi: 10.1371/journal.pone.0120091 (PMC4364902; doi:10.1371/journal.pone.0120091)

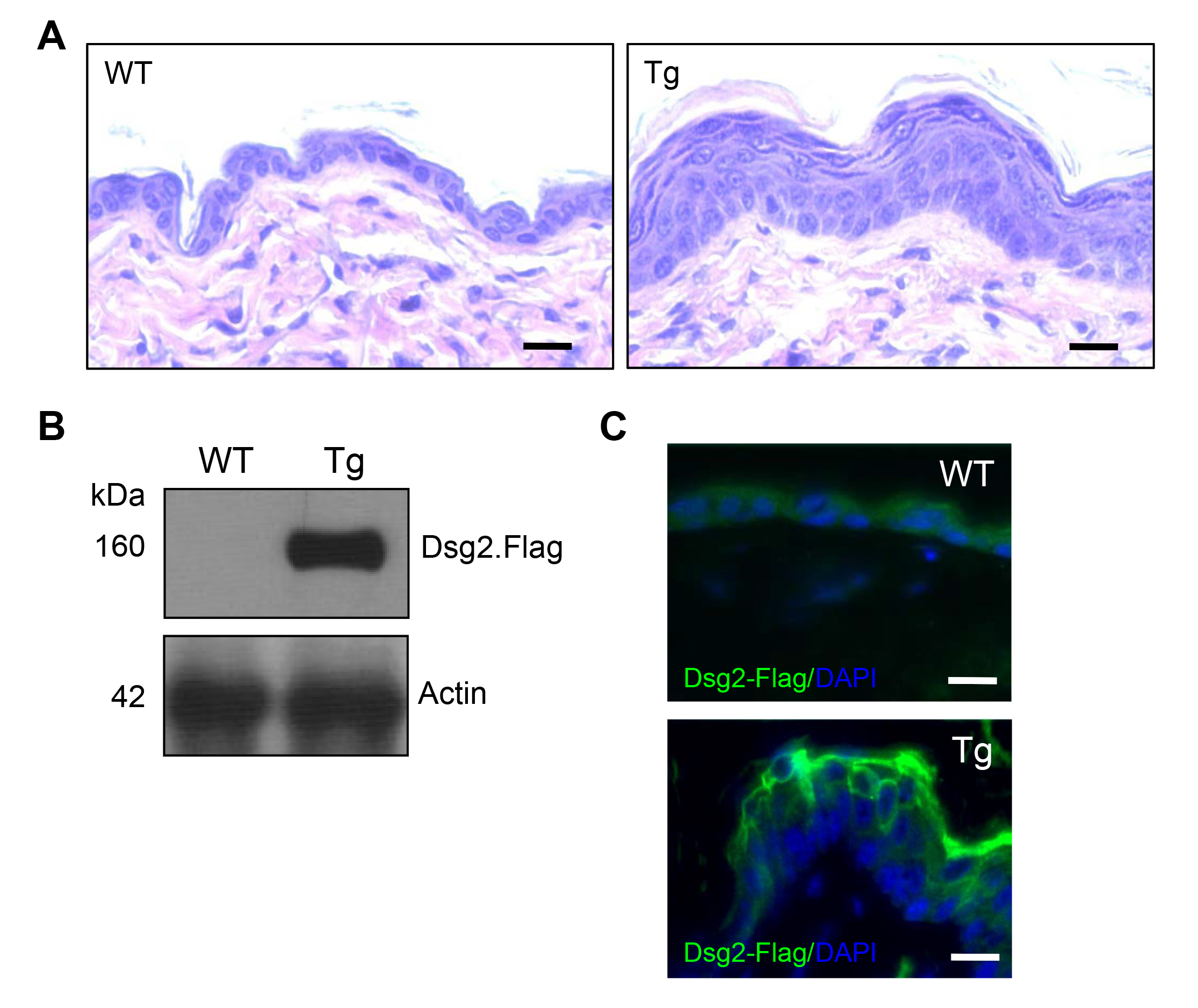

Supplement: S1 Fig — (A) H&E-staining shows epidermal hyperplasia in the Inv-Dsg2 transgenic skin compared to wild-type control. (B) Western blot analysis of Flag shows the Flag-tagged Dsg2 in the transgenic but not wild-type skin. Immunoblot with anti-Actin antibody served as loading control for protein lysates. (C) Immunofluorescent analysis reveals expression of Dsg2 in the differentiated layers of the transgenic epidermis. Nuclei were stained with DAPI (blue). Scale bar, 200 μm. Tg, transgenic; WT, wild-type. (TIF) [file pone.0120091.s001.tif]

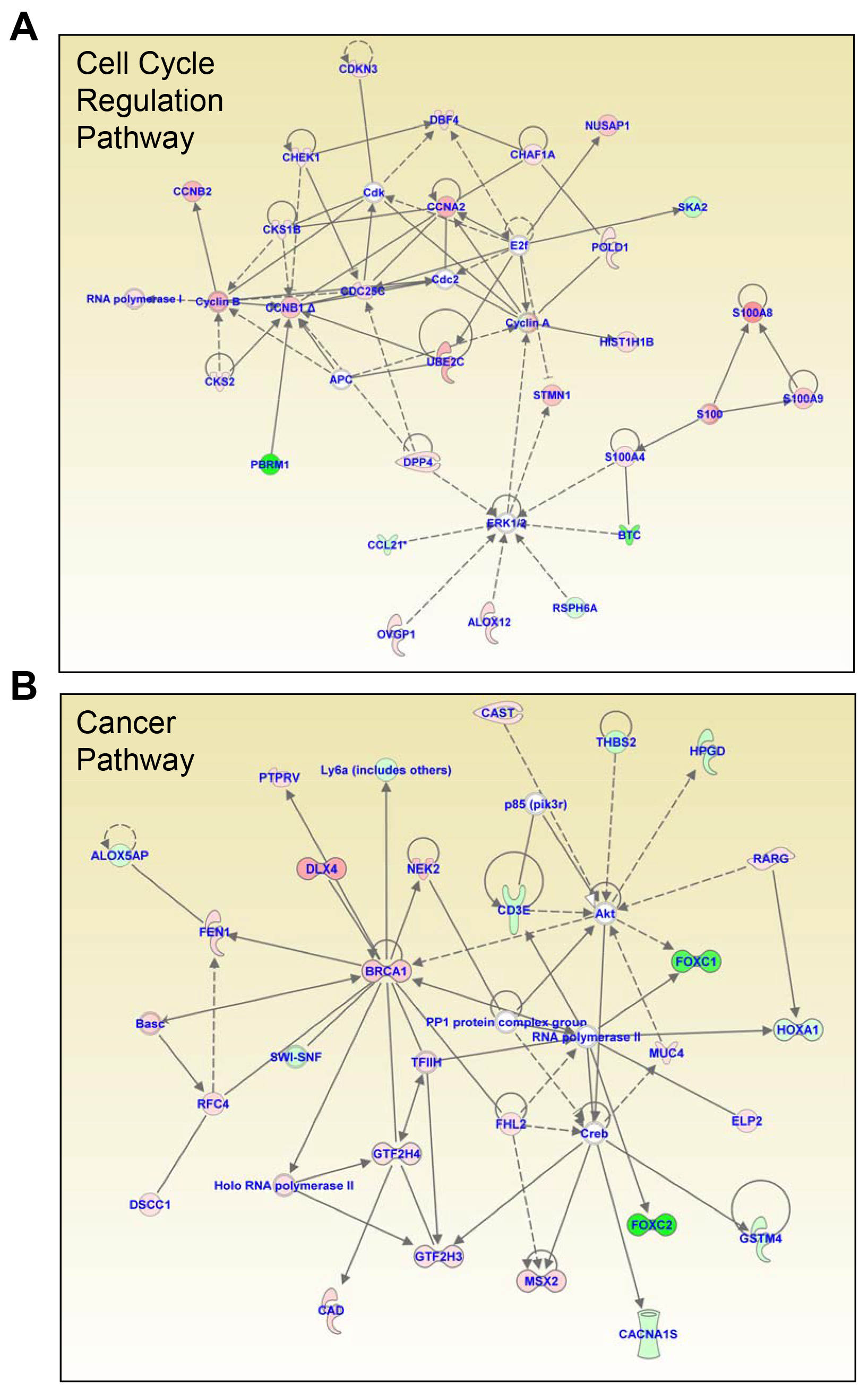

Supplement: S2 Fig — The IPA analysis revealed the top functional gene networks to be cell-cycle (A) and cancer (B) composed of multiple genes, many of which are involved in skin cancers (Cyclins, S100 family, FOXC1/2 and BRCA1 genes) to be most differentially expressed by Dsg2 compared to wild-type. Nodes represent genes and their level of color intensity is related to its level of expression (red, up-regulation; green, down-regulation). Uncolored nodes means these genes were not identified as differentially expressed and were integrated as part of the network analysis based on the information in the IPA databases. (TIF) [file pone.0120091.s002.tif]

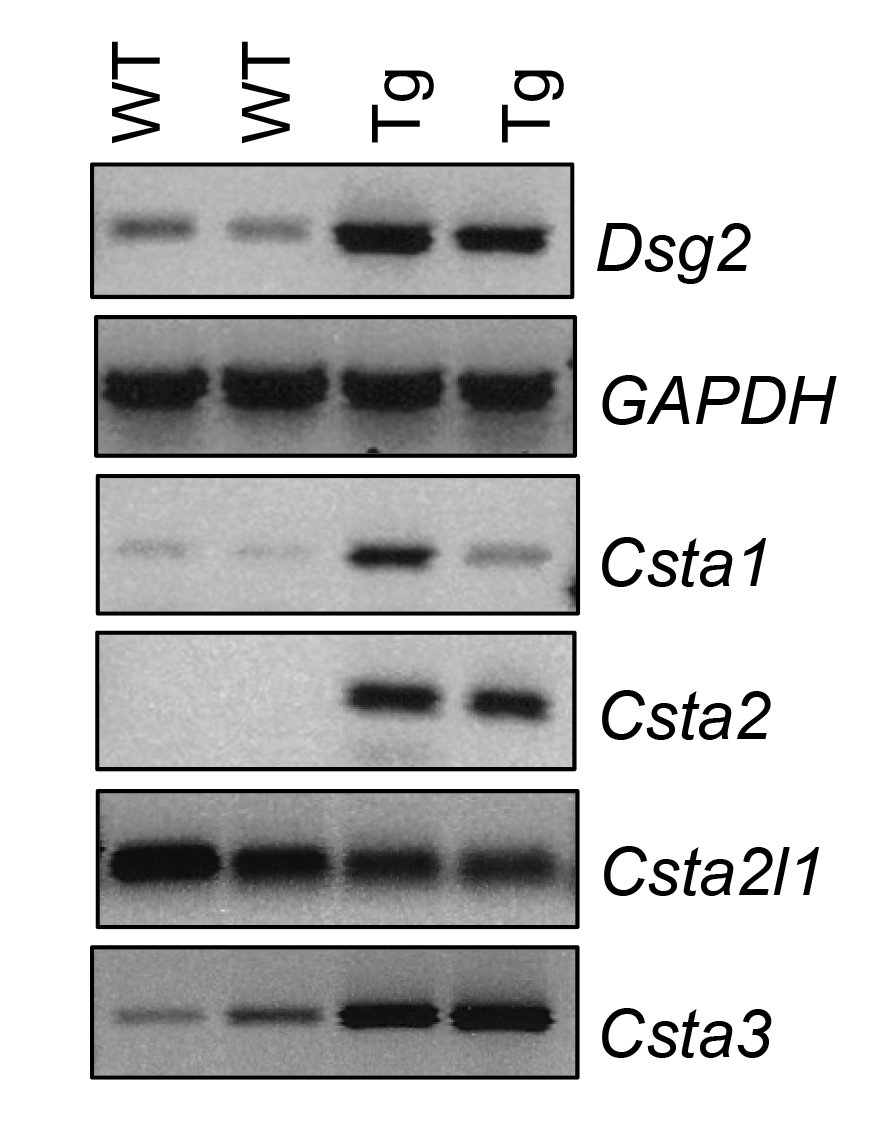

Supplement: S3 Fig — RT-PCR showed that mRNA expression of Dsg2, Csta1, Csta2 and Csta3 were relatively higher in the Inv-Dsg2 transgenic skin compared to that of wild-type. Csta2l1 expression was used as a control. (TIF) [file pone.0120091.s003.tif]

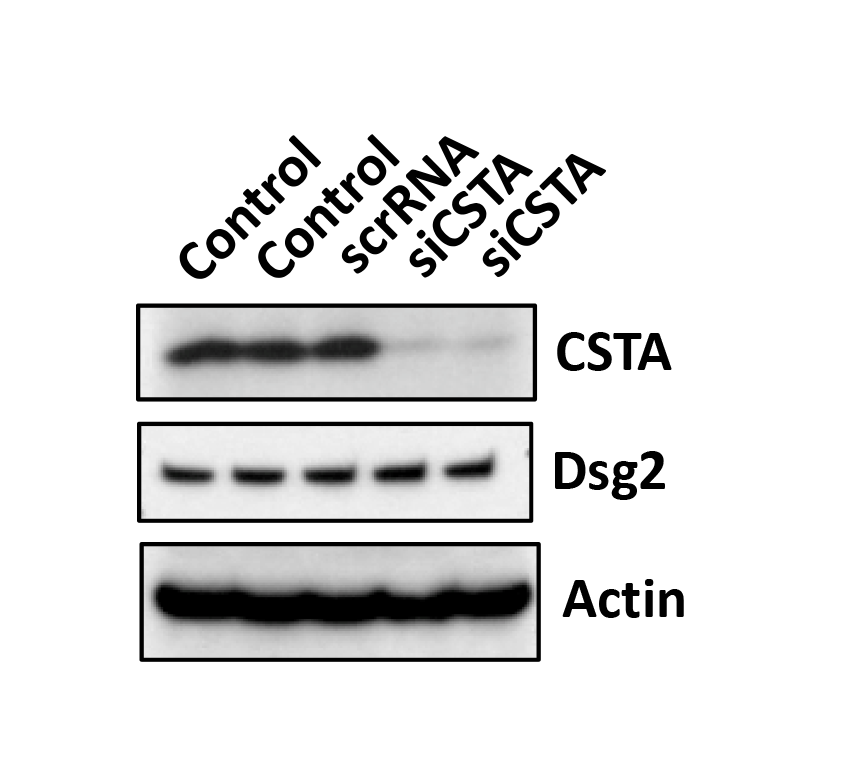

Supplement: S4 Fig — A431 cells were treated for 72 hr with 100 nM of scrambled RNA or CSTA siRNA and total protein lysate was immunoblotted for Dsg2 showing that knockdown of CSTA had no effect on Dsg2 expression. (TIF) [file pone.0120091.s004.tif]
