# Supplementary material for: Cell Cycle- and Cancer-Associated Gene Networks Activated by Dsg2: Evidence of Cystatin A Deregulation and a Potential Role in Cell-Cell Adhesion
Source: PLoS One. 2015 Mar 18;10(3):e0120091. doi: 10.1371/journal.pone.0120091 (PMC4364902; doi:10.1371/journal.pone.0120091)
Supplement: S1 Table — (PDF) [file pone.0120091.s005.pdf]

Table S1. Dsg2-dependent Gene Changes

GenBank #	Gene symbol	Gene Name	p-value	Fold
NM_021480	*TDH*	L-threonine dehydrogenase	8.1E-03	39.18
M92417	*Csta1/Stfa1*	Cystatin A1/Stefin A1	1.7E-02	18.39
D45850	*AKR1C4*	Aldo-keto reductase family 1, member C4	1.1E-02	15.18
AK007978	Unknown	Unidentified EST	3.7E-02	14.06
M92419	*Csta3/Stfa3*	Cystatin A3/Stefin A3	1.7E-02	12.37
AJ251685	*GPNMB*	Glycoprotein (transmembrane) nmb	9.1E-03	9.09
NM_009638	*CRISP3*	Cysteine-rich secretory protein 3	2.9E-02	8.79
M83218	*S100A8*	S100 calcium binding protein A8	2.2E-02	7.86
AK010010	Unknown	Unidentified EST	4.8E-02	7.40
AJ237585	*Ncapg*	Non-SMC condensin I complex, subunit G	1.3E-02	7.39
X82786	*MKI67*	Antigen identified by monoclonal antibody Ki-67	1.1E-03	7.27
AC079832	Unknown	Unidentified EST	4.3E-02	7.24
NM_019450	*IL1F6*	Interleukin 1 family, member 6 (epsilon)	3.5E-02	7.17
NM_026473	*TUBB6*	Tubulin, beta 6	1.4E-02	6.93
AK016955	Unknown	Unidentified EST	3.1E-02	6.36
NM_011472	*SPRR2A*	Small proline-rich protein 2A	8.8E-04	6.34
NM_007867	*DLX4*	Distal-less homeobox 4	5.7E-03	6.26
NM_009828	*CCNA2*	Cyclin A2	2.1E-02	5.83
Z47778	*TM4SF4*	Transmembrane 4 L six family member 4	1.2E-02	5.79
NM_008709	*MYCN*	V-myc myelocytomatosis viral related oncogene	2.6E-02	5.78
AF002823	*BUB1*	Budding uninhibited by benzimidazoles 1	1.0E-02	5.73
M92418	*Csta2/Stfa2*	Cystatin A2/Stefin A2	2.6E-02	5.71
NM_013707	*Krtap14*	Keratin associated protein 14	2.7E-03	5.46
AK021322	Unknown	Unidentified EST	5.3E-03	5.40
NM_007630	*CCNB2*	Cyclin B2	1.7E-02	5.40
AK003722	*UBE2C*	Ubiquitin-conjugating enzyme E2C	3.4E-02	5.39
NM_011328	*SCT*	Secretin	6.5E-03	4.90
AK003637	Unknown	Unidentified EST	1.2E-02	4.81
NM_009689	*BIRC5*	Baculoviral IAP repeat-containing 5	1.8E-02	4.79
NM_018815	*NUP210*	Nucleoporin 210 kDa	1.3E-02	4.71
NM_011474	*SPRR2A*	Small proline-rich protein 2A	1.2E-02	4.68
NM_026351	*Ttc39d*	Tetratricopeptide repeat domain 39D	2.1E-02	4.61
M16449	*MYB*	V-myb myeloblastosis viral oncogene homolog	2.3E-02	4.59
NM_007629	*CCNB1*	Cyclin B1	1.7E-02	4.59
NM_010790	*MELK*	Maternal embryonic leucine zipper kinase	2.6E-02	4.50
NM_019641	*STMN1*	Stathmin 1	1.7E-02	4.46
NM_011234	*RAD51*	RAD51 homolog	6.8E-03	4.34
AB041882	*PBK*	PDZ binding kinase	2.3E-02	4.31
NM_010892	*NEK2*	NIMA (never in mitosis gene a)-related kinase 2	2.3E-03	4.31
NM_016880	*KRT35*	Keratin 35	3.0E-02	4.25
AK002480	*CTH*	Cystathionase (cystathionine gamma-lyase)	3.8E-02	4.22
U85511	*NME1*	Non-metastatic cells 1, protein (NM23A)	1.1E-02	4.22
AF305710	*NUSAP1*	Nucleolar and spindle associated protein 1	6.9E-03	4.19
AK013638	*SLC39A10*	Solute carrier family 39 (zinc transporter), member 10	8.6E-03	4.12
NM_026507	*ZWILCH*	Zwilch, kinetochore associated	1.1E-02	4.11
NM_009114	*S100A9*	S100 calcium binding protein A9	5.0E-02	4.08
NM_026410	*CDCA5*	Cell division cycle associated 5	2.0E-02	4.02
AK009193	*C2orf54*	Chromosome 2 open reading frame 54	4.0E-02	3.99
AK018295	*JAKMIP2*	Janus kinase and microtubule interacting protein 2	3.8E-02	3.99
NM_021351	*CRYBA4*	Crystallin, beta A4	2.8E-02	3.98
NM_033175	*LCE3B*	Late cornified envelope 3B	5.4E-03	3.96
NM_010670	*KRTAP12-2*	Keratin associated protein 12-2	2.3E-02	3.94
U36475	*BRCA1*	Breast cancer 1, early onset	1.1E-02	3.91
NM_011524	Unknown	Unidentified EST	3.2E-02	3.87
NM_010192	*FEM1A*	Fem-1 homolog a	2.2E-02	3.86
NM_016748	*CTPS*	CTP synthase	4.8E-02	3.84
AY029584	*STEAP1*	Six transmembrane epithelial antigen of the prostate 1	1.2E-02	3.79

NM_009773	*BUB1B*	Budding uninhibited by benzimidazoles 1 homolog beta	3.4E-04	3.78
BC003428	*ASF1B*	ASF1 anti-silencing function 1 homolog B	7.9E-03	3.77
NM_009004	*KIF20A*	Kinesin family member 20A	2.2E-02	3.77
NM_021099	*KIT*	V-kit Hardy-Zuckerman 4 feline sarcoma viral oncogene	6.2E-03	3.73
AK003193	*TTYH2*	Tweety homolog 2	2.4E-02	3.71
NM_012012	*EXO1*	Exonuclease 1	4.5E-02	3.71
NM_026412	*C15orf23*	Chromosome 15 open reading frame 23	7.9E-04	3.70
NM_008970	*PTHLH*	Parathyroid hormone-like hormone	1.7E-02	3.64
AK010821	*FAM49A*	Family with sequence similarity 49, member A	3.3E-02	3.60
NM_009579	*SLC30A1*	Solute carrier family 30 (zinc transporter), member 1	2.4E-02	3.59
NM_015755	*HUNK*	Hormonally up-regulated Neu-associated kinase	4.6E-02	3.59
AK011279	*C1orf135*	Chromosome 1 open reading frame 135	1.2E-02	3.58
AK009086	*KRTAP26-1*	Keratin associated protein 26-1	4.0E-02	3.53
NM_008252	*HMGB2*	High-mobility group box 2	4.4E-04	3.52
NM_011369	*SHCBP1*	SHC SH2-domain binding protein 1	1.8E-02	3.49
AK010336	*TRIP13*	Thyroid hormone receptor interactor 13	5.9E-03	3.46
U68495	Unknown	Unidentified EST	4.6E-03	3.42
AY029203	*B3GNT5*	UDP-GlcNAc:betaGal beta-1,3-N-acetylglucosaminyltransferase 5	3.2E-02	3.41
NM_011404	*SLC7A5*	Solute carrier family 7, member 5	3.6E-02	3.37
NM_007681	*CENPA*	Centromere protein A	5.0E-03	3.35
NM_008217	*HAS3*	Hyaluronan synthase 3	1.3E-03	3.35
NM_008566	*MCM5*	Minichromosome maintenance complex component 5	1.8E-02	3.34
NM_013905	*HEYL*	Hairy/enhancer-of-split related with YRPW motif-like	1.4E-02	3.34
NM_021380	*IL20*	Interleukin 20	2.9E-02	3.32
U79738	*DLX3*	Distal-less homeobox 3	9.7E-03	3.26
AB037685	*ANP32E*	Acidic (leucine-rich) nuclear phosphoprotein 32 family, member E	3.4E-02	3.23
NM_010665	Unknown	Unidentified EST	2.0E-02	3.22
M94303	Unknown	Unidentified EST	1.1E-02	3.19
M30774	Unknown	Unidentified EST	2.1E-02	3.17
NM_009860	*CDC25C*	Cell division cycle 25 homolog C	1.7E-02	3.16
NM_021790	*CENPK*	Centromere protein K	3.0E-02	3.11
NM_009705	*ARG2*	Arginase, type II	2.3E-02	3.10
NM_016889	*INSM1*	Insulinoma-associated 1	2.0E-02	3.10
NM_008253	*HMGB3*	High-mobility group box 3	1.1E-02	3.09
NM_020567	*GMNN*	Geminin, DNA replication inhibitor	4.0E-02	3.09
AK005954	*SYCE2*	Synaptonemal complex central element protein 2	4.5E-02	3.08
AK012894	*ZNF618*	Zinc finger protein 618	1.8E-02	3.07
AK011728	*SPC24*	SPC24, NDC80 kinetochore complex component	2.9E-02	3.06
J03733	*ODC1*	Ornithine decarboxylase 1	4.67E-04	3.06
AF053339	*CAD*	Carbamoyl-phosphate synthetase 2, aspartate transcarbamylase, and dihydroorotase	4.7E-02	3.04
NM_013726	*DBF4*	DBF4 homolog	1.1E-02	3.03
NM_016692	*INCENP*	Inner centromere protein antigens 135/155kDa	9.6E-03	2.99
NM_012044	*PLA2G2E*	Phospholipase A2, group IIE	3.7E-02	2.98
AK008278	*CKAP2L*	Cytoskeleton associated protein 2-like	9.2E-03	2.95
NM_013601	*MSX2*	Msh homeobox 2	2.9E-02	2.95
NM_019754	*TAGLN3*	Transgelin 3	1.1E-02	2.95
D10920	*ZNF618*	Zinc finger protein 618	2.6E-02	2.91
NM_009028	*Rasl2-9-ps*	RAS-like, family 2, locus 9, pseudogene	1.1E-02	2.91
AK003555	*SGOL2*	Shugoshin-like 2	1.5E-02	2.88
AK004134	*ANLN*	Anillin, actin binding protein	2.1E-02	2.84
X62154	*MCM3*	Minichromosome maintenance complex component 3	3.2E-02	2.83
NM_007999	*FEN1*	Flap structure-specific endonuclease 1	1.6E-02	2.81
NM_008239	*FOXQ1*	Forkhead box Q1	3.9E-02	2.80
NM_008565	*MCM4*	Minichromosome maintenance complex component 4	4.6E-02	2.79
NM_008580	*MAP3K5*	Mitogen-activated protein kinase kinase kinase 5	3.4E-02	2.78
X90779	Unknown	Unidentified EST	2.5E-03	2.76
NM_009171	*SHMT1*	Serine hydroxymethyltransferase 1 (soluble)	1.4E-02	2.75
AF091101	*DUT*	Deoxyuridine triphosphatase	2.8E-02	2.74
AK015015	*STEAP2*	Six transmembrane epithelial antigen of the prostate 2	4.1E-03	2.74

NM_021352	*CRYBB3*	Crystallin, beta B3	4.6E-02	2.74
NM_028186	*NKD2*	Naked cuticle homolog 2	3.6E-02	2.73
BC011230	*NT5DC2*	5'-nucleotidase domain containing 2	8.0E-04	2.72
AK021390	*HELLS*	Helicase, lymphoid-specific	2.2E-03	2.71
BC003335	*RFC4*	Replication factor C (activator 1) 4, 37kDa	4.1E-02	2.69
NM_007440	*ALOX12*	Arachidonate 12-lipoxygenase	9.9 E-03	2.69
NM_008128	*GJB6*	Gap junction protein, beta 6, 30kDa	4.0E-02	2.68
AK009031	Unknown	Unidentified EST	6.3E-04	2.64
AK010426	*CDKN3*	Cyclin-dependent kinase inhibitor 3	3.0E-02	2.64
NM_009015	*RAD54L*	RAD54-like	1.6E-02	2.64
AK011596	*TFRC*	Transferrin receptor (p90, CD71)	3.2E-02	2.63
AK020710	Unknown	Unidentified EST	3.5E-02	2.63
NM_007955	*PTPRV*	Protein tyrosine phosphatase, receptor type, V, pseudogene	6.1E-03	2.63
NM_009128	*Scd2*	Stearoyl-Coenzyme A desaturase 2	1.1E-02	2.63
AK009787	*IL1F8*	Interleukin 1 family, member 8 (eta)	3.5E-02	2.62
U62922	*HIST1H1B*	Histone cluster 1, H1b	3.1E-03	2.62
AK013046	Unknown	Unidentified EST	2.1E-02	2.61
NM_016870	*ACSM3*	Acyl-CoA synthetase medium-chain family member 3	4.1E-03	2.61
U26229	Unknown	Unidentified EST	3.4E-02	2.61
AK008384	Unknown	Unidentified EST	2.3E-02	2.59
NM_007633	*CCNE1*	Cyclin E1	3.3E-03	2.59
NM_010715	*LIG1*	Ligase I, DNA, ATP-dependent	3.2E-02	2.55
AK009799	*NOP56*	NOP56 ribonucleoprotein homolog	1.6E-02	2.53
M62766	*HMGCR*	3-hydroxy-3-methylglutaryl-CoA reductase	1.9E-02	2.53
NM_015774	*ERO1L*	ERO1-like	9.4E-04	2.52
AK003919	*SP6*	Sp6 transcription factor	1.5E-02	2.51
AK004208	*C1orf128*	Chromosome 1 open reading frame 128	1.1E-02	2.51
BC005610	*TADA2A*	Transcriptional adaptor 2A	6.4E-03	2.51
NM_021308	*PIWIL2*	Piwi-like 2	1.6E-03	2.50
AK003197	*KTI12*	KTI12 homolog, chromatin associated	3.9E-02	2.48
NM_011514	*SUV39H1*	Suppressor of variegation 3-9 homolog 1	1.5E-03	2.48
NM_018736	*MRE11A*	MRE11 meiotic recombination 11 homolog A	3.5E-02	2.47
AK013659	*ARMC1*	Armadillo repeat containing 1	1.6E-02	2.46
NM_026394	*Lce1f*	Late cornified envelope 1F	1.5E-02	2.45
X80433	*CIRH1A*	Cirrhosis, autosomal recessive 1A	1.6E-02	2.45
AK012015	*EXOSC1*	Exosome component 1	2.8E-02	2.43
AK014047	*LIPG*	Lipase, endothelial	2.3E-02	2.43
NM_007494	*ASS1*	Argininosuccinate synthase 1	1.8E-03	2.42
NM_010074	*DPP4*	Dipeptidyl-peptidase 4	5.7E-03	2.42
AK011410	*FAM54A*	Family with sequence similarity 54, member A	2.3E-02	2.41
BC005751	*SDAD1*	SDA1 domain containing 1	1.6E-02	2.41
NM_025578	*MRPS25*	Mitochondrial ribosomal protein S25	1.5E-02	2.41
NM_008684	*NEO1*	Neogenin 1	1.7E-02	2.38
NM_011060	*PADI3*	Peptidyl arginine deiminase, type III	4.0E-02	2.38
NM_020506	*XPO4*	Exportin 4	9.6E-03	2.38
U69106	*AURKA*	Aurora kinase A	1.6E-04	2.38
AK004331	*PPIL1*	Peptidylprolyl isomerase (cyclophilin)-like 1	3.5E-02	2.37
AK012041	*CDT1*	Chromatin licensing and DNA replication factor 1	4.8E-02	2.37
AK017176	*GTF2H3*	General transcription factor IIH, polypeptide 3, 34kDa	8.3E-03	2.37
AK004509	*ASPSCR1*	Alveolar soft part sarcoma chromosome region, candidate 1	3.0E-02	2.35
NM_018868	*NOP58*	NOP58 ribonucleoprotein homolog	2.0E-02	2.35
NM_011391	*SLC16A7*	Solute carrier family 16, member 7	2.3E-02	2.34
AY007202	*MUC4*	Mucin 4, cell surface associated	2.0E-02	2.32
NM_016710	*HMGN5*	High-mobility group nucleosome binding domain 5	3.9E-02	2.32
AK004699	*CYP2S1*	Cytochrome P450, family 2, subfamily S, polypeptide 1	1.6E-03	2.31
NM_008652	*MYBL2*	V-myb myeloblastosis viral oncogene homolog-like 2	5.9E-03	2.31
NM_010212	*FHL2*	Four and a half LIM domains 2	4.9E-03	2.31
NM_018749	*EIF3D*	Eukaryotic translation initiation factor 3, subunit D	4.2E-02	2.30
NM_026531	*AEN*	Apoptosis enhancing nuclease	9.6E-03	2.29

NM_031863	*CENPQ*	Centromere protein Q	3.7E-02	2.29
Z31362	*SRXN1*	Sulfiredoxin 1	4.0E-02	2.29
AK010786	*TUBB2B*	Tubulin, beta 2B	1.1E-03	2.28
AK021069	*S100A4*	S100 calcium binding protein A4	2.2E-02	2.28
NM_019800	*ACP6*	Acid phosphatase 6, lysophosphatidic	5.0E-02	2.28
NM_020487	*PRSS21*	Protease, serine, 21 (testisin)	2.2E-02	2.28
NM_026041	*RRP15*	Ribosomal RNA processing 15 homolog	1.4E-02	2.28
X54327	*EPRS*	Glutamyl-prolyl-tRNA synthetase	1.7E-02	2.28
NM_008062	*G6PD*	Glucose-6-phosphate dehydrogenase	1.9E-02	2.26
NM_016924	*RWDD2B*	RWD domain containing 2B	4.1E-02	2.26
NM_019451	*IL1F5*	Interleukin 1 family, member 5 (delta)	2.6E-02	2.26
NM_025415	*CKS2*	CDC28 protein kinase regulatory subunit 2	3.3E-02	2.25
S70056	*CRYZ*	Crystallin, zeta (quinone reductase)	1.4E-02	2.24
X65089	Unknown	Unidentified EST	3.3E-02	2.24
NM_010053	*DLX1*	Distal-less homeobox 1	3.5E-02	2.23
NM_010066	*DNMT1*	DNA (cytosine-5-)-methyltransferase 1	1.2E-02	2.22
AK014598	*PEX26*	Peroxisomal biogenesis factor 26	2.6E-02	2.21
AK016519	*CCDC18*	Coiled-coil domain containing 18	1.6E-03	2.21
D14336	*POLR1E*	Polymerase (RNA) I polypeptide E, 53kDa	2.1E-02	2.21
NM_007573	*C1QBP*	Complement component 1, q subcomponent binding protein	3.9E-03	2.21
NM_015782	*SNRPA*	Small nuclear ribonucleoprotein polypeptide A	2.8E-03	2.21
NM_016693	*MAP3K6*	Mitogen-activated protein kinase kinase kinase 6	1.5E-02	2.21
NM_019468	*G6pd2*	Glucose-6-phosphate dehydrogenase 2	2.6E-02	2.21
AK005294	*PRPF31*	PRP31 pre-mRNA processing factor 31 homolog	5.0E-02	2.20
NM_016904	*CKS1B*	CDC28 protein kinase regulatory subunit 1B	2.8E-02	2.20
NM_026024	*UBE2T*	Ubiquitin-conjugating enzyme E2T (putative)	4.3E-03	2.20
AK016940	*FGD6*	FYVE, RhoGEF and PH domain containing 6	2.1E-02	2.19
BC013242	*FOS*	FBJ murine osteosarcoma viral oncogene homolog	3.6E-02	2.19
NM_010792	*METTL1*	Methyltransferase like 1	2.9E-02	2.19
NM_011239	*RANBP1*	RAN binding protein 1	3.0E-02	2.19
AK012621	Unknown	Unidentified EST	3.5E-02	2.18
NM_009270	*SQLE*	Squalene epoxidase	1.3E-03	2.18
NM_011244	*RARG*	Retinoic acid receptor, gamma	1.8E-02	2.18
NM_013733	*CHAF1A*	Chromatin assembly factor 1, subunit A (p150)	4.2E-02	2.18
NM_013903	*MMP20*	Matrix metallopeptidase 20	3.4E-02	2.18
AF075717	*Tiaf2*	TGF-beta1-induced anti-apoptotic factor 2	4.7E-02	2.17
AK012157	*Unknown*	Unidentified EST	4.9E-02	2.17
AK020373	*Unknown*	Unidentified EST	3.0E-02	2.16
NM_009521	*WNT3*	Wingless-type MMTV integration site family, member 3	3.5E-02	2.16
U62675	Unknown	Unidentified EST	1.5E-02	2.16
AB025582	*ANP32B*	Acidic (leucine-rich) nuclear phosphoprotein 32 family, member B	1.2E-02	2.15
AF251276	*SERPINI2*	Serpin peptidase inhibitor, clade I (pancpin), member 2	3.0E-02	2.15
AK011162	*DSCC1*	Defective in sister chromatid cohesion 1 homolog	5.5E-03	2.15
X53584	*HSPD1*	Heat shock 60kDa protein 1 (chaperonin)	4.8E-03	2.15
NM_019412	*PRX*	Periaxin	1.3E-02	2.14
AF120322	Unknown	Unidentified EST	4.5E-02	2.13
AK002481	*PAQR5*	Progestin and adipoQ receptor family member V	1.7E-02	2.13
AK011820	*FBXO5*	F-box protein 5	4.9E-02	2.13
BC010981	*BC010981*	cDNA sequence BC010981	2.7E-02	2.13
NM_010722	*LMNB2*	Lamin B2	4.7E-04	2.13
NM_011171	*PROCR*	Protein C receptor, endothelial	1.7E-02	2.13
AB026997	*CAST*	Calpastatin	3.5E-02	2.12
AB041663	*CCDC86*	Coiled-coil domain containing 86	8.4E-03	2.12
AK004150	Unknown	Unidentified EST	2.9E-02	2.12
AB015613	*SET*	SET nuclear oncogene	3.6E-02	2.11
AK008621	*C11orf90*	Chromosome 11 open reading frame 90	1.3E-02	2.11
D32137	*OVGP1*	Oviductal glycoprotein 1, 120kDa	4.7E-02	2.11
NM_010364	*GTF2H4*	General transcription factor IIH, polypeptide 4, 52kDa	2.6E-02	2.11
NM_030004	*CRYL1*	Crystallin, lambda 1	2.9E-02	2.11

AK008369	*Snhg8*	Small nucleolar RNA host gene 8	1.3E-03	2.10
AK012762	Unknown	Unidentified EST	4.9E-02	2.10
BC003426	*EIF2B1*	Eukaryotic translation initiation factor 2B, subunit 1 alpha	3.5E-02	2.10
L36062	*STAR*	Steroidogenic acute regulatory protein	3.4E-02	2.10
NM_007832	*DCK*	Deoxycytidine kinase	2.0E-02	2.10
AK021362	*FAM55D*	Family with sequence similarity 55, member D	1.7E-02	2.09
AB007600	*CYBRD1*	Cytochrome b reductase 1	1.3E-02	2.08
NM_007691	*CHEK1*	CHK1 checkpoint homolog	4.0E-02	2.08
NM_009982	*CTSC*	Cathepsin C	2.3E-02	2.08
Y18276	*NBEA*	Neurobeachin	2.0E-02	2.08
AK018070	*LPO*	Lactoperoxidase	1.4E-02	2.07
NM_008180	*GSS*	Glutathione synthetase	2.6E-02	2.07
NM_021336	*SNRPA1*	Small nuclear ribonucleoprotein polypeptide A'	3.9E-02	2.07
NM_025403	*NOP10*	NOP10 ribonucleoprotein homolog	4.4E-02	2.07
AF293845	*LPAR3*	Lysophosphatidic acid receptor 3	4.5E-02	2.06
AK020690	*Krtap5-5*	Keratin associated protein 5-5	5.0E-02	2.06
NM_011568	*THOC4*	THO complex 4	2.8E-02	2.06
NM_016777	*NASP*	Nuclear autoantigenic sperm protein (histone-binding)	1.2E-02	2.06
NM_009088	*POLR1A*	Polymerase (RNA) I polypeptide A, 194kDa	2.2E-03	2.05
NM_016788	*TNK2*	Tyrosine kinase, non-receptor, 2	2.1E-02	2.05
NM_019740	*FOXO3*	Forkhead box O3	3.7E-02	2.05
AK012340	*NMRAL1*	NmrA-like family domain containing 1	4.3E-02	2.04
NM_007926	*AIMP1*	Aminoacyl tRNA synthetase complex-interacting multifunctional protein 1	2.9E-03	2.04
NM_008424	*KCNE1*	Potassium voltage-gated channel, Isk-related family, member 1	2.7E-02	2.04
NM_011131	*POLD1*	Polymerase (DNA directed), delta 1, catalytic subunit 125kDa	1.5E-02	2.04
AF343088	*KRT75*	Keratin 75	1.7E-02	2.03
AK018333	*PWP2*	PWP2 periodic tryptophan protein homolog	3.3E-02	2.03
NM_008300	*HSPA4*	Heat shock 70 kDa protein 4	2.7E-04	2.03
NM_008893	*POLA2*	Polymerase (DNA directed), alpha 2 (70 kD subunit)	3.9E-03	2.02
AB003502	*GSPT1*	G1 to S phase transition 1	1.6E-02	2.01
AK002371	*SNRNP40*	Small nuclear ribonucleoprotein 40 kDa (U5)	1.1E-02	2.01
AK010531		Unidentified EST	3.3E-02	2.01
AK011725	*APITD1*	Apoptosis-inducing, TAF9-like domain 1	2.3E-03	2.01
NM_009104	*RRM2*	Ribonucleotide reductase M2	4.7E-02	2.01
NM_015765	*HSPA14*	Heat shock 70kDa protein 14	1.7E-02	2.01
NM_015781	*NAP1L1*	Nucleosome assembly protein 1-like 1	2.1E-02	2.01
NM_020603	*WDR46*	WD repeat domain 46	3.6E-03	2.01
NM_021448	*ELP2*	Elongation protein 2 homolog	1.0E-02	2.01
NM_023136	*DTYMK*	Deoxythymidylate kinase (thymidylate kinase)	3.2E-02	2.01
NM_026306	*TRMT112*	tRNA methyltransferase 11-2 homolog	6.1E-03	2.01
NM_011304	*RUVBL2*	RuvB-like 2	8.8E-03	2.00
NM_025424	*NENF*	Neuron derived neurotrophic factor	8.0E-03	-2.00
AK011423	*GNS*	Glucosamine (N-acetyl)-6-sulfatase	5.0E-03	-2.01
Z31360	Unknown	Unidentified EST	2.3E-02	-2.01
NM_007628	*CCNA1*	Cyclin A1	2.9E-02	-2.02
AF398966	*ASB5*	Ankyrin repeat and SOCS box-containing 5	4.3E-02	-2.03
AK019470	*GALNTL2*	UDP-N-acetyl-alpha-D-galactosamine:polypeptide N-acetylgalactosaminyltransferase-like 2	3.9E-02	-2.03
NM_013630	*PKD1*	Polycystic kidney disease 1 (autosomal dominant)	1.5E-02	-2.03
M33425	*JAK1*	Janus kinase 1	1.7E-02	-2.04
NM_008495	*LGALS1*	Lectin, galactoside-binding, soluble, 1	3.3E-02	-2.04
NM_008816	*PECAM1*	Platelet/endothelial cell adhesion molecule	3.0E-02	-2.04
NM_016985	*MTMR1*	Myotubularin related protein 1	9.3E-03	-2.04
NM_025425	*RPL3L*	Ribosomal protein L3-like	3.1E-02	-2.04
AF373409	*Cd209b*	CD209b antigen	2.9E-02	-2.05
AK009522	*C9orf25*	Chromosome 9 open reading frame 25	5.0E-02	-2.05
NM_010449	*HOXA1*	Homeobox A1	1.6E-03	-2.05
NM_026514	*CDC42EP3*	CDC42 effector protein (Rho GTPase binding) 3	1.8E-02	-2.06
NM_031255	*RSPH6A*	Radial spoke head 6 homolog A (Chlamydomonas)	1.6E-02	-2.06

U12147	*LAMA2*	Laminin, alpha 2	4.5E-02	-2.06
BC012232	*S1PR5*	Sphingosine-1-phosphate receptor 5	1.5E-02	-2.07
NM_016872	*VAMP5*	Vesicle-associated membrane protein 5 (myobrevin)	3.1E-02	-2.07
AK018685	*TTC39B*	Tetratricopeptide repeat domain 39B	3.6E-02	-2.08
AY042200	*MRGPRX1*	MAS-related GPR, member X1	2.8E-03	-2.08
BC012207	Unknown	Unidentified EST	2.4E-02	-2.08
AF115383	*DST*	Dystonin	4.3E-03	-2.09
BC006062	*ANO1*	Anoctamin 1, calcium activated chloride channel	2.0E-02	-2.09
NM_010231	*FMO1*	Flavin containing monooxygenase 1	1.8E-02	-2.09
NM_021371	*CALN1*	Calneuron 1	2.0E-02	-2.09
AF322069	*TNFRSF21*	Tumor necrosis factor receptor superfamily, member 21	1.9E-02	-2.10
NM_008768	*ORM1/ORM2*	Orosomucoid 1	2.2E-02	-2.11
AK004559	*CPNE8*	Copine VIII	4.3E-02	-2.12
AK018343	*SYT11*	Synaptotagmin XI	2.6E-02	-2.12
AK019934	*CNRIP1*	Cannabinoid receptor interacting protein 1	4.0E-03	-2.12
NM_008764	*TNFRSF11B*	Tumor necrosis factor receptor superfamily, member 11b	8.6E-03	-2.12
NM_010361	*GSTT2/GSTT2B*	Glutathione S-transferase theta 2	3.1E-02	-2.12
NM_019919	*LTBP1*	Latent transforming growth factor beta binding protein 1	1.0E-02	-2.12
NM_019965	*DNAJB12*	DnaJ (Hsp40) homolog, subfamily B, member 12	4.9E-02	-2.12
U50959	*PLCB4*	Phospholipase C, beta 4	3.9E-03	-2.12
AK009757	*PIR*	Pirin (iron-binding nuclear protein)	1.2E-02	-2.13
AK009847	*PRSS23*	Protease, serine, 23	3.4E-02	-2.13
AK012007	Unknown	Unidentified EST	3.0E-03	-2.13
NM_008505	*LMO2*	LIM domain only 2 (rhombotin-like 1)	1.3E-03	-2.13
NM_008867	*PLA2R1*	Phospholipase A2 receptor 1, 180kDa	7.9E-03	-2.13
BC006619	Unknown	Unidentified EST	2.6E-02	-2.14
AF414190	*SLC35B2*	Solute carrier family 35, member B2	4.8E-02	-2.15
AK007469	*ABHD14B*	Abhydrolase domain containing 14B	1.3E-03	-2.15
AK010042	*SPINK5*	Serine peptidase inhibitor, Kazal type 5	1.5E-02	-2.15
AK015409	Unknown	Unidentified EST	4.1E-02	-2.15
NM_008885	*PMP22*	Peripheral myelin protein 22	3.6E-02	-2.15
AF133093	Unknown	Unidentified EST	3.8E-02	-2.16
AF161262	*TFAP4*	Transcription factor AP-4 (activating enhancer binding protein 4)	2.0E-02	-2.16
AK004726	*LYVE1*	Lymphatic vessel endothelial hyaluronan receptor 1	3.7E-02	-2.16
AK005457	*CBX7*	Chromobox homolog 7	1.9E-02	-2.16
NM_008908	*PPIC*	Peptidylprolyl isomerase C (cyclophilin C)	3.6E-02	-2.16
BC006902	*SLC46A3*	Solute carrier family 46, member 3	3.0E-02	-2.17
AL357453	*EID1*	EP300 interacting inhibitor of differentiation 1	3.5E-02	-2.18
BC005485	*OLFML3*	Olfactomedin-like 3	2.0E-02	-2.18
NM_020265	*DKK2*	Dickkopf homolog 2	1.0E-02	-2.18
AK004365	Unknown	Unidentified EST	9.0E-04	-2.19
AK018282	*FAM81A*	Family with sequence similarity 81, member A	1.0E-02	-2.19
X65506	*KRT36*	Keratin 36	1.6E-02	-2.20
AK007436	*ADAMTS9*	ADAM metallopeptidase with thrombospondin type 1 motif, 9	4.6E-03	-2.21
AK010208	*APOL6*	Apolipoprotein L, 6	2.7E-02	-2.21
AK014014	Unknown	Unidentified EST	2.6E-03	-2.21
AK015592	Unknown	Unidentified EST	4.2E-02	-2.22
NM_020591	Unknown	Unidentified EST	2.5E-02	-2.22
AF336850	*Cyp2j9*	Cytochrome P450, family 2, subfamily j, polypeptide 9	4.6E-02	-2.23
AK004681	*TRIL*	TLR4 interactor with leucine-rich repeats	4.8E-02	-2.23
NM_010630	*KIFC2*	Kinesin family member C2	2.2E-02	-2.23
AK020118	*GPC6*	Glypican 6	9.5E-03	-2.24
BC004727	*VWA5A*	Von Willebrand factor A domain containing 5A	4.3E-02	-2.26
M22479	*TPM1*	Tropomyosin 1 (alpha)	1.4E-02	-2.26
M96554	*ALOX5AP*	Arachidonate 5-lipoxygenase-activating protein	8.3E-03	-2.26
NM_010654	*KLRD1*	Killer cell lectin-like receptor subfamily D, member 1	1.1E-02	-2.26
Z47769	Unknown	Unidentified EST	5.4E-04	-2.26
AK013704	Unknown	Unidentified EST	1.3E-02	-2.27
BC008560	*SSFA2*	Sperm specific antigen 2	4.4E-02	-2.27

NM_008508	*LOR*	Loricrin	1.1E-02	-2.27
NM_016687	*SFRP4*	Secreted frizzled-related protein 4	4.8E-02	-2.27
NM_017378	*PCDH12*	Protocadherin 12	3.6E-02	-2.27
AF248643	*ACTN2*	Actinin, alpha 2	5.0E-02	-2.28
AK003626	*SLC38A4*	Solute carrier family 38, member 4	3.6E-03	-2.28
AK013968	Unknown	Unidentified EST	1.8E-02	-2.28
NM_020291	*OR5P3*	Olfactory receptor, family 5, subfamily P, member 3	3.3E-02	-2.28
AK006564	Unknown	Unidentified EST	9.3E-04	-2.32
NM_010276	*GEM*	GTP binding protein overexpressed in skeletal muscle	6.8 E-03	-2.32
AF235001	*SLC5A5*	Solute carrier family 5 (sodium iodide symporter), member 5	2.3E-02	-2.33
NM_010741	*Ly6a*	Lymphocyte antigen 6 complex, locus A	1.2E-02	-2.33
AK002587	*ASPDH*	Aspartate dehydrogenase domain containing	7.8E-03	-2.35
BC010828	*DAP*	Death-associated protein	8.6E-03	-2.35
NM_009608	*ACTC1*	Actin, alpha, cardiac muscle 1	5.2E-03	-2.35
NM_010330	*EMB*	Embigin	2.8E-02	-2.35
NM_025658	*Ms4a4c*	Membrane-spanning 4-domains, subfamily A, member 4C	3.6E-02	-2.35
AK004184	*NEXN*	Nexilin (F actin binding protein)	2.6E-02	-2.36
J05479	*PPP3CA*	Protein phosphatase 3, catalytic subunit, alpha isozyme	3.7E-02	-2.36
NM_027218	*Clec4b1*	C-type lectin domain family 4, member b1	1.4E-02	-2.36
AK010184	Unknown	Unidentified EST	1.7E-02	-2.37
U20264	*Copg2as2*	Coatomer protein complex, subunit gamma 2, antisense 2	4.1E-03	-2.37
AK018860	Unknown	Unidentified EST	5.6E-03	-2.38
NM_021416	*FAM184B*	Family with sequence similarity 184, member B	3.1E-02	-2.38
NM_016894	*RAMP1*	Receptor (G protein-coupled) activity modifying protein 1	3.7E-02	-2.39
AK010044	Unknown	Unidentified EST	4.0E-02	-2.40
NM_007470	*APOD*	Apolipoprotein D	1.9E-02	-2.41
NM_013589	*LTBP2*	Latent transforming growth factor beta binding protein 2	1.4E-02	-2.41
NM_021472	*RNASE4*	Ribonuclease, RNase A family, 4	4.4E-02	-2.42
AK017323	*TPRG1*	Tumor protein p63 regulated 1	4.1E-03	-2.43
NM_007409	*ADH1C*	Alcohol dehydrogenase 1C (class I), gamma polypeptide	1.6E-02	-2.43
NM_008969	*PTGS1*	Prostaglandin-endoperoxide synthase 1	4.8E-02	-2.43
NM_023052	*CCL21*	Chemokine (C-C motif) ligand 21	6.9E-03	-2.43
AK010153	*TTN*	Titin	2.9E-02	-2.44
M23013	*TF*	Transferrin	1.6E-03	-2.44
NM_021543	*PCDH8*	Protocadherin 8	4.6E-02	-2.44
AK003418	*GSTM4*	Glutathione S-transferase mu 4	3.2E-02	-2.47
NM_008409	*ITM2A*	Integral membrane protein 2A	4.2E-02	-2.48
AY033514	*BBOX1*	Butyrobetaine (gamma), 2-oxoglutarate dioxygenase (gamma-butyrobetaine hydroxylase) 1	2.3E-02	-2.49
NM_009349	*INMT*	Indolethylamine N-methyltransferase	1.4E-02	-2.50
NM_014193	*CACNA1S*	Calcium channel, voltage-dependent, L type, alpha 1S subunit	1.2E-02	-2.51
AK009124	Unknown	Unidentified EST	3.4E-02	-2.52
AK015595	Unknown	Unidentified EST	1.8E-02	-2.52
NM_013553	*HOXC4*	Homeobox C4	4.8E-02	-2.52
Z25851	Unknown	Unidentified EST	2.1E-02	-2.53
AK005515	*C3orf59*	Chromosome 3 open reading frame 59	3.7E-02	-2.54
NM_015803	*ATP8A2*	ATPase, aminophospholipid transporter, class I, type 8A, member 2	2.6E-02	-2.54
AK004155	*THEM5*	Thioesterase superfamily member 5	3.5 E-02	-2.56
AK006219	Unknown	Unidentified EST	5.3E-03	-2.57
NM_010484	*SLC6A4*	Solute carrier family 6, member 4	3.7E-02	-2.57
AF203899	*NEB*	Nebulin	3.3E-02	-2.58
NM_011620	*TNNT3*	Troponin T type 3 (skeletal, fast)	3.0E-02	-2.59
AK016603	*TKTL2*	Transketolase-like 2	1.2E-02	-2.60
AK017769	*CCDC41*	Coiled-coil domain containing 41	4.6E-02	-2.60
AK009330	*CLDN23*	Claudin 23	1.4E-02	-2.62
BC004653	*SMARCA2*	SWI/SNF related, matrix associated, actin dependent regulator of chromatin, subfamily a, member 2	4.0E-02	-2.62
NM_021422	*DNAJA4*	DnaJ (Hsp40) homolog, subfamily A, member 4	4.8E-02	-2.63
U70380	Unknown	Unidentified EST	8.7E-03	-2.64
AK010217	Unknown	Unidentified EST	4.6E-02	-2.65

AK003535	*TMEM140*	Transmembrane protein 140	1.3E-02	-2.66
NM_009263	*SPP1*	Secreted phosphoprotein 1	1.5E-02	-2.66
AK003496	*FAM98C*	Family with sequence similarity 98, member C	9.5E-03	-2.67
NM_021282	*CYP2E1*	Cytochrome P450, family 2, subfamily E, polypeptide 1	3.1E-02	-2.67
AB020886	*AKAP12*	A kinase (PRKA) anchor protein 12	2.8E-02	-2.69
NM_007421	*ADSSL1*	Adenylosuccinate synthase like 1	3.2E-02	-2.69
AY042202	*Mrgprb4/Mrgprb5*	MAS-related GPR, member B5	1.5E-02	-2.71
NM_011335	*CCL21*	Chemokine (C-C motif) ligand 21	2.2E-02	-2.72
NM_007728	*COCH*	Coagulation factor C homolog, cochlin (Limulus polyphemus)	2.9E-02	-2.75
NM_026667	*FAM114A1*	Family with sequence similarity 114, member A1	3.5E-02	-2.75
AK014194	*RHOBTB1*	Rho-related BTB domain containing 1	2.8E-03	-2.76
AK009197	*APBB2*	Amyloid beta (A4) precursor protein-binding, family B, member 2	2.7E-03	-2.77
AK013631	*TPPP*	tubulin polymerization promoting protein	4.3E-02	-2.77
AK017349	Unknown	Unidentified EST	1.6E-02	-2.77
AK021161	*WDR65*	WD repeat domain 65	2.0E-03	-2.81
NM_008455	*KLKB1*	Kallikrein B, plasma (Fletcher factor) 1	1.1E-02	-2.81
AK019879	Unknown	Unidentified EST	2.0E-02	-2.84
AK009816	*TRDN*	Triadin	1.9E-02	-2.85
AB048364	*GDPD2*	Glycerophosphodiester phosphodiesterase domain containing 2	1.7E-03	-2.86
AK019750	Unknown	Unidentified EST	2.2E-02	-2.87
NM_009849	*ENTPD2*	Ectonucleoside triphosphate diphosphohydrolase 2	1.1E-02	-2.87
Z12270	Unknown	Unidentified EST	7.6E-03	-2.90
NM_019759	*DPT*	Dermatopontin	1.1E-03	-2.91
NM_022982	*RTN4R*	Reticulon 4 receptor	1.2E-02	-2.91
NM_009943	*COX6A2*	Cytochrome c oxidase subunit VIa polypeptide 2	1.1E-02	-2.92
AK008747	*NFATC1*	Nuclear factor of activated T-cells, cytoplasmic, calcineurin-dependent 1	9.8E-03	-2.93
NM_011281	*RORC*	RAR-related orphan receptor C	2.4E-02	-2.93
NM_007648	*CD3E*	CD3e molecule, epsilon (CD3-TCR complex)	2.2E-02	-2.95
NM_008278	*HPGD*	Hydroxyprostaglandin dehydrogenase 15-(NAD)	3.4E-02	-2.99
Z95477	Unknown	Unidentified EST	1.9E-03	-3.00
NM_013711	*TXNRD2*	Thioredoxin reductase 2	3.2E-03	-3.04
NM_008086	*GAS1*	Growth arrest-specific 1	1.6E-02	-3.05
AK016066	Unknown	Unidentified EST	3.3E-02	-3.06
NM_007933	*ENO3*	Enolase 3 (beta, muscle)	3.1E-02	-3.07
AF141322	*CAV2*	Caveolin 2	1.7E-03	-3.08
NM_008047	*FSTL1*	Follistatin-like 1	2.8E-02	-3.08
AK012513	Unknown	Unidentified EST	1.9E-02	-3.09
NM_007813	*Cyp2b13/Cyp2b9*	Cytochrome P450, family 2, subfamily b, polypeptide 9	4.0E-03	-3.11
NM_011224	*PYGM*	Phosphorylase, glycogen, muscle	1.3E-04	-3.13
NM_011581	*THBS2*	Thrombospondin 2	1.3E-02	-3.13
BC003847	*IL33*	Interleukin 33	4.3E-02	-3.14
X73020	Unknown	Unidentified EST	5.1E-03	-3.15
NM_010555	*IL1R2*	Interleukin 1 receptor, type II	1.6E-02	-3.21
AK003152	*TTN*	Titin	2.6E-02	-3.27
NM_007453	*PRDX6*	Peroxiredoxin 6	1.0E-02	-3.27
AK003046	*NRN1*	Neuritin 1	5.5E-03	-3.28
NM_021508	*MYOZ1*	Myozenin 1	4.9E-02	-3.29
NM_032541	*HAMP*	Hepcidin antimicrobial peptide	1.8E-02	-3.29
NM_011867	*SLC26A4*	Solute carrier family 26, member 4	3.2E-02	-3.36
AK017827	*EIF2C4*	Eukaryotic translation initiation factor 2C, 4	2.4E-02	-3.39
NM_010195	*LGR5*	Leucine-rich repeat-containing G protein-coupled receptor 5	3.7E-02	-3.40
NM_025377	*SKA2*	Spindle and kinetochore associated complex subunit 2	9.5E-03	-3.41
AJ005051	*Gm10883*	Predicted gene 10883	4.8E-02	-3.47
NM_010758	*MAG*	Myelin associated glycoprotein	1.3E-02	-3.47
NM_010834	*MSTN*	Myostatin	1.8E-02	-3.65
NM_010244	*Fv1*	Friend virus susceptibility 1	4.0E-03	-3.70
NM_007802	*CTSK*	Cathepsin K	4.6E-02	-3.88
BC014684	*SLC7A13*	Solute carrier family 7, (cationic amino acid transporter, y+ system) member 13	1.1E-02	-3.95

NM_011580	*THBS1*	Thrombospondin 1	4.7E-02	-4.02
NM_013645	*PVALB*	Parvalbumin	3.8E-03	-4.17
NM_019548	*TRO*	Trophinin	1.1E-03	-4.58
AK013470	Unknown	Unidentified EST	4.5E-03	-4.64
NM_013467	*ALDH1A1*	Aldehyde dehydrogenase 1 family, member A1	4.5E-02	-4.69
NM_019564	*HTRA1*	HtrA serine peptidase 1	3.5E-03	-4.73
NM_009394	*TNNC2*	Troponin C type 2 (fast)	7.3E-03	-4.78
AK009107	*XIRP2*	Xin actin-binding repeat containing 2	1.8E-02	-4.88
AK014524	*SERPINB7*	Serpin peptidase inhibitor, clade B (ovalbumin), member 7	8.6E-03	-4.88
AY042201	*Mrgprb3*	MAS-related GPR, member B3	8.8E-03	-4.89
NM_007538	*OPN1SW*	Opsin 1 (cone pigments), short-wave-sensitive	1.1E-02	-4.93
X78885	*HNRNPA1*	Heterogeneous nuclear ribonucleoprotein A1	2.8E-03	-4.97
NM_019687	*SLC22A4*	Solute carrier family 22 (organic cation/ergothioneine transporter), member 4	2.1E-03	-5.07
NM_008343	*IGFBP3*	Insulin-like growth factor binding protein 3	3.4E-03	-5.24
NM_026535	*SERPINA12*	Serpin peptidase inhibitor, clade A (alpha-1 antiproteinase, antitrypsin), member 12	8.1E-03	-5.84
NM_008791	*PCP4*	Purkinje cell protein 4	3.9E-03	-6.58
AK019744	Unknown	Unidentified EST	6.1E-03	-6.84
AF070470	*SMOC1*	SPARC related modular calcium binding 1	4.1E-02	-6.89
NM_007568	*BTC*	Betacellulin	1.0E-02	-7.75
AK018865	Unknown	Unidentified EST	1.3E-02	-8.22
NM_008592	*FOXC1*	Forkhead box C1	3.3 E-02	-8.95
BC014714	*HMGCS2*	3-hydroxy-3-methylglutaryl-CoA synthase 2 (mitochondrial)	1.3 E-02	-8.98
NM_013519	*FOXC2*	Forkhead box C2 (MFH-1, mesenchyme forkhead 1)	3.8E-02	-11.69
AK004289	Unknown	Unidentified EST	8.3E-03	-25.73
AK009582	*PBRM1*	Polybromo 1	5.1E-03	-32.82

Complete list of 492 genes modified greater or less than 2 fold in response to Dsg2 expression in transgenic mice.
