# Supplementary material for: Cell Cycle- and Cancer-Associated Gene Networks Activated by Dsg2: Evidence of Cystatin A Deregulation and a Potential Role in Cell-Cell Adhesion
Source: PLoS One. 2015 Mar 18;10(3):e0120091. doi: 10.1371/journal.pone.0120091 (PMC4364902; doi:10.1371/journal.pone.0120091)
Supplement: S2 Table — (PDF) [file pone.0120091.s006.pdf]

Table S2. Associated Network Functions

**Top 5 Gene Networks**
Gene Expression, Cell Cycle, Cancer
Embryonic Development, Tissue Development, Cancer
Cell Cycle, Cancer, Genetic Disorder
Cellular Assembly and Organization, DNA Replication, Recombination, and Repair, Cell Cycle
Dermatological Diseases and Conditions, Genetic Disorder, Tissue Morphology
