# Supplementary material for: Cell Cycle- and Cancer-Associated Gene Networks Activated by Dsg2: Evidence of Cystatin A Deregulation and a Potential Role in Cell-Cell Adhesion
Source: PLoS One. 2015 Mar 18;10(3):e0120091. doi: 10.1371/journal.pone.0120091 (PMC4364902; doi:10.1371/journal.pone.0120091)
Supplement: S3 Table — (PDF) [file pone.0120091.s007.pdf]

Table S3. Changes in Expression of Cell Cycle Genes in Response to Dsg2

G1 Phase and G1/S Transition:<br>Camk2a, Camk2b, Gpr132, Itgb1, Mtbp, Myb (C-MYB), **Nfatc1 (-2.9)**, Ppp2r3a, **Ppp3ca (-2.4)**, Skp2, Taf10, Slfn1
S Phase and DNA Replication:<br>Dnajc2 (Zrf2), Mcm2, **Mcm3 (2.8)**, **Mcm4 (2.8)**, **Mki67 (7.8)**, **Mre11a (2.5)**, **Msh2 (2.9)**, Pcna, Rad17, **Rad51 (4.3)**, Sumo1.
G2 Phase and G2/M Transition:<br>Dnajc2 (Zrf2), **Chek1 (2.1)**, Ppm1d.
M Phase:<br>**Brca2 (3.9)**, **Ccna1 (Cyclin A1, -2.0)**, **Ccnb1 (4.6)**, Cdc25a, Cdc25b, Cdk2, Nek2, Npm2, Pes1, Prm1, Rad21, Ran, Shc1, Smc1a (Smc1l1), Stag1, Terf1, Tnfsf5ip1, Wee1.
Cell Cycle Checkpoint and Cell Cycle Arrest:<br>Ak1, Apbb1, **Brca2 (3.9)**, Casp3, Cdk5rap1, Cdkn1a, Cdkn1b, Cdkn2a, **Chek1 (2.1)**, **Cks1b (2.1)**, Ddit3 (CHOP), **Dst (-2.1)**, Gadd45a, Hus1, Inha, Macf1, Mad2l1, Mdm2, **Msh2 (2.9)**, Notch2, **Pkd1 (-2.1)**, **Pmp22 (-2.1)**, Ppm1d (WIP1), Rad9, Sesn2 (Sestrin 2), Sfn (Stratifin), Slfn1, Smc1a (Smc1l1), Tsg101.
Regulation of the Cell Cycle:<br>Abl1, Brca2, **Ccna1 (-2.0)**, **Ccna2 (5.8)**, **Ccnb1 (4.6)**, **Ccnb2 (5.4)**, Ccnc, Ccnd1, **Ccne1 (2.6)**, Ccnf, Cdk4, Cdkn1a, Cks1b, E2f1, E2f2, E2f3, E2f4, Gadd45a, Itgb1, Rad9, Ran, Sfn (Stratifin), Shc1, Skp2, Tfdp1, Tnfsf5ip1.
Negative Regulation of the Cell Cycle:<br>Apbb1, Atm, **Brca1 (3.9)**, Casp3, Cdkn2a, Inha, Rbl1, Rbl2, Trp53, Trp63.

Listed are genes known to be involved in different phases of cell cycle regulation. In bold are genes with change greater, or less, than 2 fold in Dsg2 transgenic compared to control wild-type mice and in parentheses are fold change.
